# New species of semi-aquatic freshwater earthworm genus *Glyphidrilus* Horst, 1889 from Thailand and Laos (Oligochaeta, Almidae)

**DOI:** 10.3897/zookeys.672.10212

**Published:** 2017-05-03

**Authors:** Ratmanee Chanabun, Khamla Inkavilay, Somsak Panha

**Affiliations:** 1 Program in Animal Science, Faculty of Agriculture Technology, Sakon Nakhon Rajabhat University, Sakon Nakhon 47000, Thailand; 2 Animal Systematics Research Unit, Department of Biology, Faculty of Science, Chulalongkorn University, Bangkok 10330, Thailand; 3 Department of Biology, Faculty of Science, National University of Laos, Vientiane, Laos P.D.R.

**Keywords:** Almidae, earthworms, *Glyphidrilus*, Laos, southeast Asia, Thailand

## Abstract

Seven new species of semi-aquatic freshwater earthworms belonging to the genus *Glyphidrilus* Horst, 1889 are described from Thailand and Laos, *Glyphidrilus nanensis* Chanabun & Panha **sp. n.**, *G. satunensis* Chanabun & Panha **sp. n.**, *G. chiangraiensis* Chanabun & Panha **sp. n.**, *G. namphao* Chanabun & Panha **sp. n.**, *G. sekongensis* Chanabun & Panha **sp. n.**, *G. namdonensis* Chanabun & Panha **sp. n.**, and *G. champasakensis* Chanabun & Panha **sp. n.** The species are characterized by their external and internal morphological characteristics, as well as body sizes. Other morphological character differences between these seven species were also compared, and an identification key is provided. The relationships of the new species to congeners are discussed.

## Introduction

The semi-aquatic freshwater earthworm genus *Glyphidrilus* Horst, 1889 has been known since the nineteenth century. The unique morphological characters of the expanded epidermis at approximately the clitellum position, called “wings”, and the absence of prostate glands, the rounded body with a posterior quadrangular shape and the long slender banana-like cocoons are prominent in *Glyphidrilus*. The semi-aquatic habitat between terrestrial and freshwater ecosystems of rivers, streams, canals, ponds, swamps or even in paddy rice systems are also consistent (Horst 1889, 1893, Jamieson 1968, Michaelsen 1896, 1897, 1900, 1902, 1910, 1918, 1922, Rao 1922, Shen and Yeo 2005, Chanabun et al. 2013). Up to now, the records of *Glyphidrilus* are only from Africa and Asia; however, most described species are from Asia and especially from Southeast Asia. The worms are now becoming threatened because of the modification, pollution, and destruction of their habitats, for example the contamination by chemical agriculture, and the dam constructions in the upper Mekong River (pers. obs.).

Most previous species were described with some illustrations in a format which was frequently poorly interpreted and insufficient in some species. However, in the recent descriptions and redescriptions of several species are mostly from Thailand, and some from Malaysia, Singapore, and Laos, these deficiencies have been corrected. The color images of both animals and habitats together with anatomical illustration details have made improvements in a new description format. The 19 newly described species reported bring the total number to 40 recognized *Glyphidrilus* species (Chanabun et al. 2011, Chanabun et al. 2012a, Chanabun et al. 2012b, Chanabun et al. 2013, Chanabun and Panha 2015, Jirapatrasilp et al. 2016).

The behavior of animals has been observed, and it was noted that the worms leave their tail tips exposed near surface of their muddy habitats. While submerging they produce casts as do most earthworms (Chanabun et al. 2013). It was also found that north of 12 degrees latitude in Thailand *Glyphidrilus* appears as mostly adults in the dry to early rainy seasons (March to July), while in the rainy season they mainly appeared as juveniles, especially in the upper parts of Thailand. However, south of 12 degrees latitude, the adults seem to be present all year round (pers. obs.).

Chanabun et al. (2013) interpreted the phylogeography of *Glyphidrilus* in light of recent hypotheses regarding ancient river drainage patterns, especially the Mekong River and other main basins, plus various habitat types, using morphological characters and genetic data. Enzyme electrophoresis has proved that the closely related species *G. mekongensis* Panha & Chanabun, 2012 and *G. vangviengensis* Panha & Chanabun, 2011 occurring along the lower Mekong River basin are definitely separate biological species; some possible cryptic species are also suggested in the paper (Jirapatrasilp et al. 2015). The present paper provides additional new species with careful morphological descriptions but no additional genetic data.

## Materials and methods

**Figure 1.**
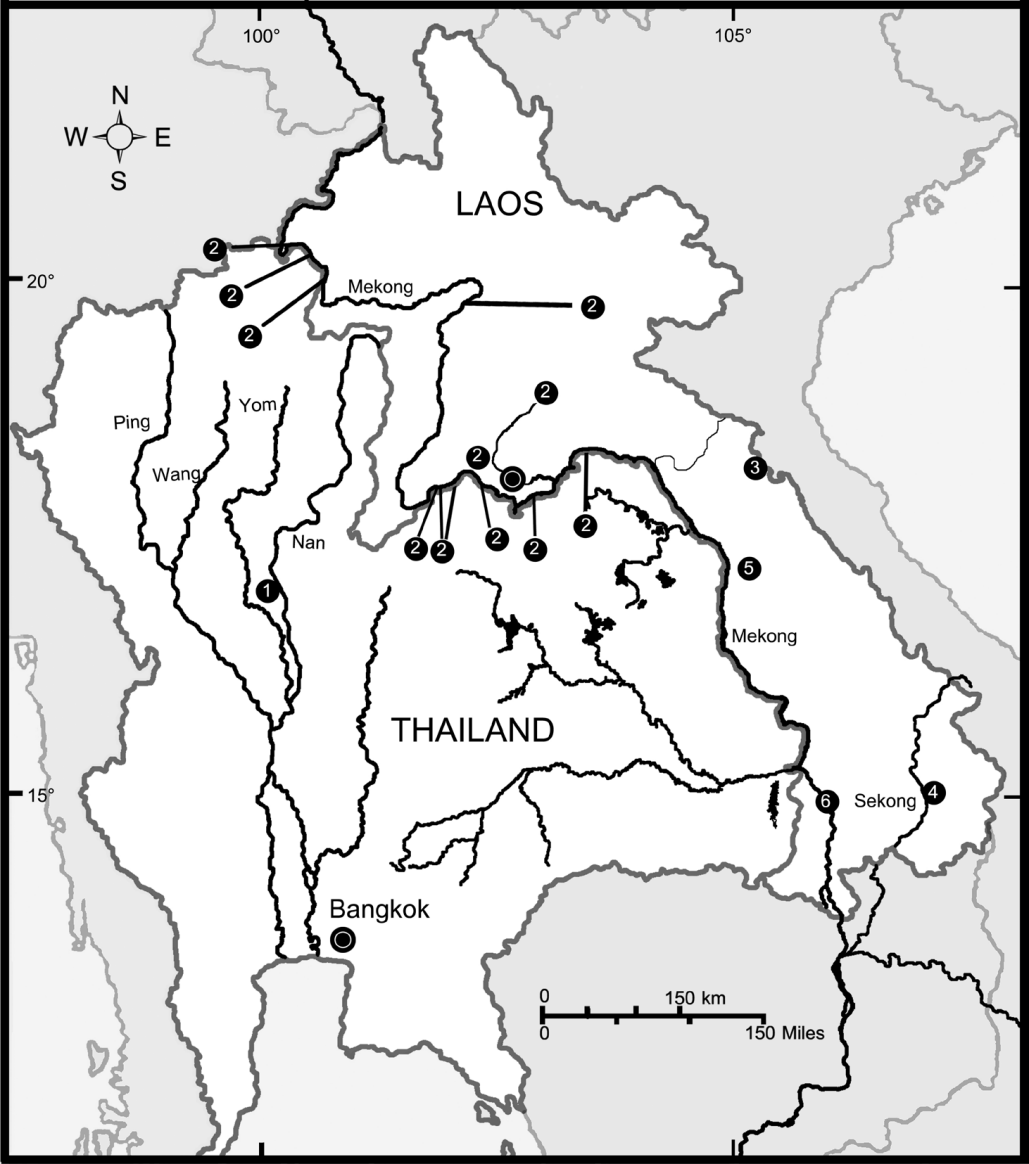
Distribution map of the new *Glyphidrilus* species from Thailand and Laos. Numbers in the circles refer to localities of the new species. **1** *Glyphidrilus nanensis* sp. n. **2** *Glyphidrilus chiangraiensis* sp. n. **3** *Glyphidrilus namphao* sp. n. **4** *Glyphidrilus sekongensis* sp. n. **5** *Glyphidrilus namdonensis* sp. n. and **6** *Glyphidrilus champasakensis* sp. n.

**Figure 2.**
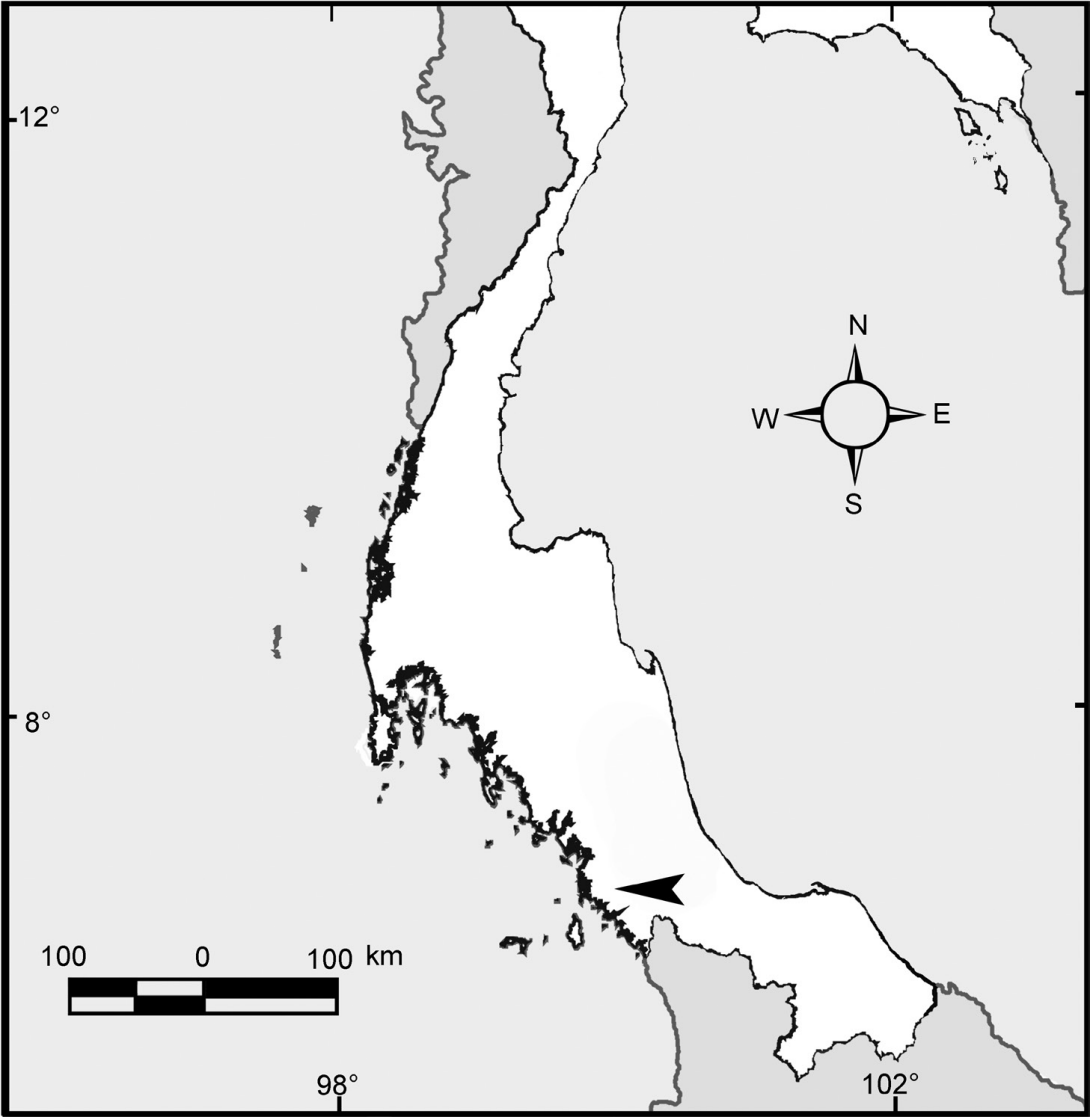
Map showing type locality of *Glyphidrilus satunensis* sp. n. (arrow head) located on the shore of the Nong Prakpraya at Mueang, Satun, south Thailand.

The systematic and faunistic surveys of *Glyphidrilus* were conducted in the lower Mekong River basin both in Thailand and Laos (Fig. 1), and some other river systems in Thailand (Fig. 2) from June 2012 to April 2014. The collections were made by carefully digging up the topsoil near casts on the shore and in the water using hand sorting and sieving the soil from river banks. Adults, juveniles, and cocoons were collected and killed in 30% (v/v) ethanol, transferred to 5% (w/v) formalin for fixation in approximately 12 hours, and then transferred to 75% (v/v) ethanol for standard preservation and subsequent morphological studies. Duplicate specimens and/or tissue samples were preserved in 95% ethanol for further molecular and DNA barcoding analyses.

The type series are deposited in the Chulalongkorn University, Museum of Zoology, Bangkok, Thailand (**CUMZ**). Additional paratypes will be deposited in the Natural History Museum (**NHMUK**), London, Raffles Museum of Biodiversity Research, National University of Singapore, Singapore (**ZRC**) and at the Biozentrum Grindel und Zoologisches Museum, University of Hamburg (**ZMH**).

The descriptions were made from observations under an Olympus SZX16 stereoscopic light microscope. The following external and internal morphological characters were investigated and recorded: body length and segment number; the positions of clitellum and clitellar wings, genital markings, intestinal origin, gizzard, spermathecae, hearts and seminal vesicles. Illustrations were made for the whole body segments and the external and internal characters. The body width and length were measured in both full adults and juveniles, and are presented as the range (min-max) and mean±one standard deviation (SD).

Comparative studies of *Glyphidrilus* type specimens were made at four natural history museums:

**CUMZ** Chulalongkorn University, Museum of Zoology.

**ZRC** Raffles Museum of Biodiversity Research, National University of Singapore, Singapore.

**NHMUK** The Natural History Museum, London.

**ZMH** Biozentrum Grindel und Zoologisches Museum, University of Hamburg, Germany.

Anatomical abbreviations are as follows (Chanabun et al. 2013):

**gm** genital markings

**he** hearts

**np** nephridia

**sc** spermathecae

**sv** seminal vesicles

**wi** wings

## Systematics

### Family ALMIDAE Duboscq, 1902

#### Genus Glyphidrilus

Taxon classificationAnimaliaOpisthoporaAlmidae

Horst, 1889

##### Diagnosis.

Prostomium zygolobous. Body shape nearly circular in cross section in anterior part, and becoming quadrangular in posterior part or after clitellum. Anus dorsal or dorso-terminal. A longitudinal lamellar ridge at maturity from body wall on each side in bc, through several of the clitellar segments, which are called wings. Dorsal pores absent. Setae four pairs per segment. Clitellum annular. Genital apertures, all minute and superficial. Male pores inter-or intraclitellar. Spermathecal pores usually all behind the testis segments. Gizzard in VII or VIII sometimes extending into an adjacent segment. Calciferous glands absent. Seminal vesicles usually short, usually four pairs in IX–XII. Holonephridia. Nephrostomes single ducts avesiculate and without sphincters or caeca. Testis and funnels free in X and XI. Male ducts intramural. Ovaries fan shaped and with several egg strings. Ovisacs present or absent. Prostate glands absent and spermathecae without diverticulum (Chanabun et al. 2013, Gates 1972).

##### Type species.

*Glyphidrilus weberi* Horst, 1889. Type species by original designation in Horst (1889: 77).

#### Glyphidrilus nanensis

Taxon classificationAnimaliaOpisthoporaAlmidae

Chanabun & Panha sp. n.

http://zoobank.org/E3CF3E43-7045-4C0F-9946-73AAB425425A

**Figure 3.**
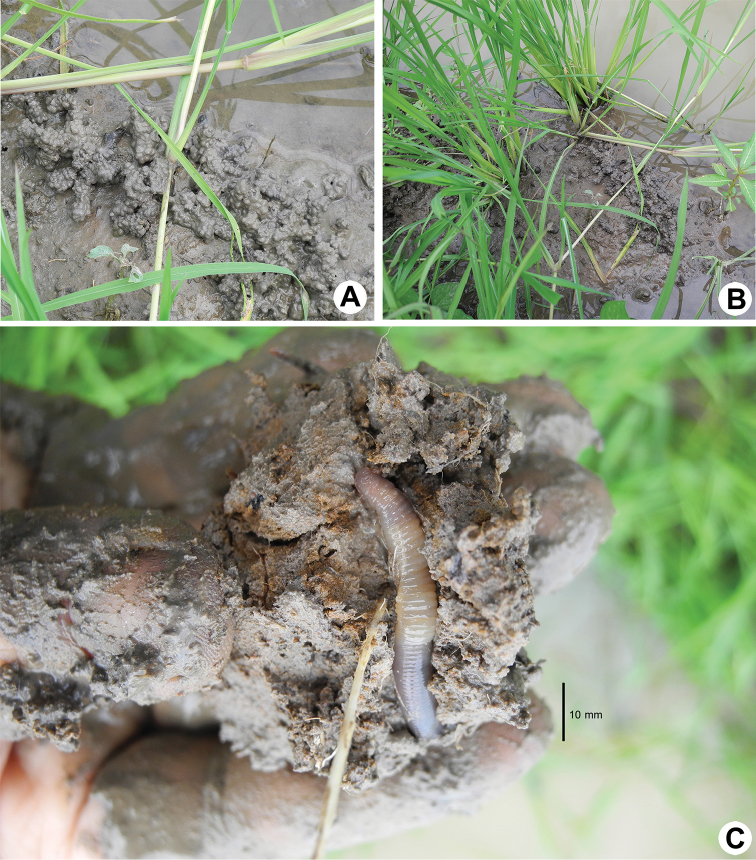
Photographs showing the **A** *Glyphidrilus nanensis* sp. n. casts **B** type locality of *G. nanensis* sp. n. in the rice field near Nan River at Saklek, Phichit, north Thailand **C** coloration of living paratype (CUMZ 3404).

**Figure 4.**
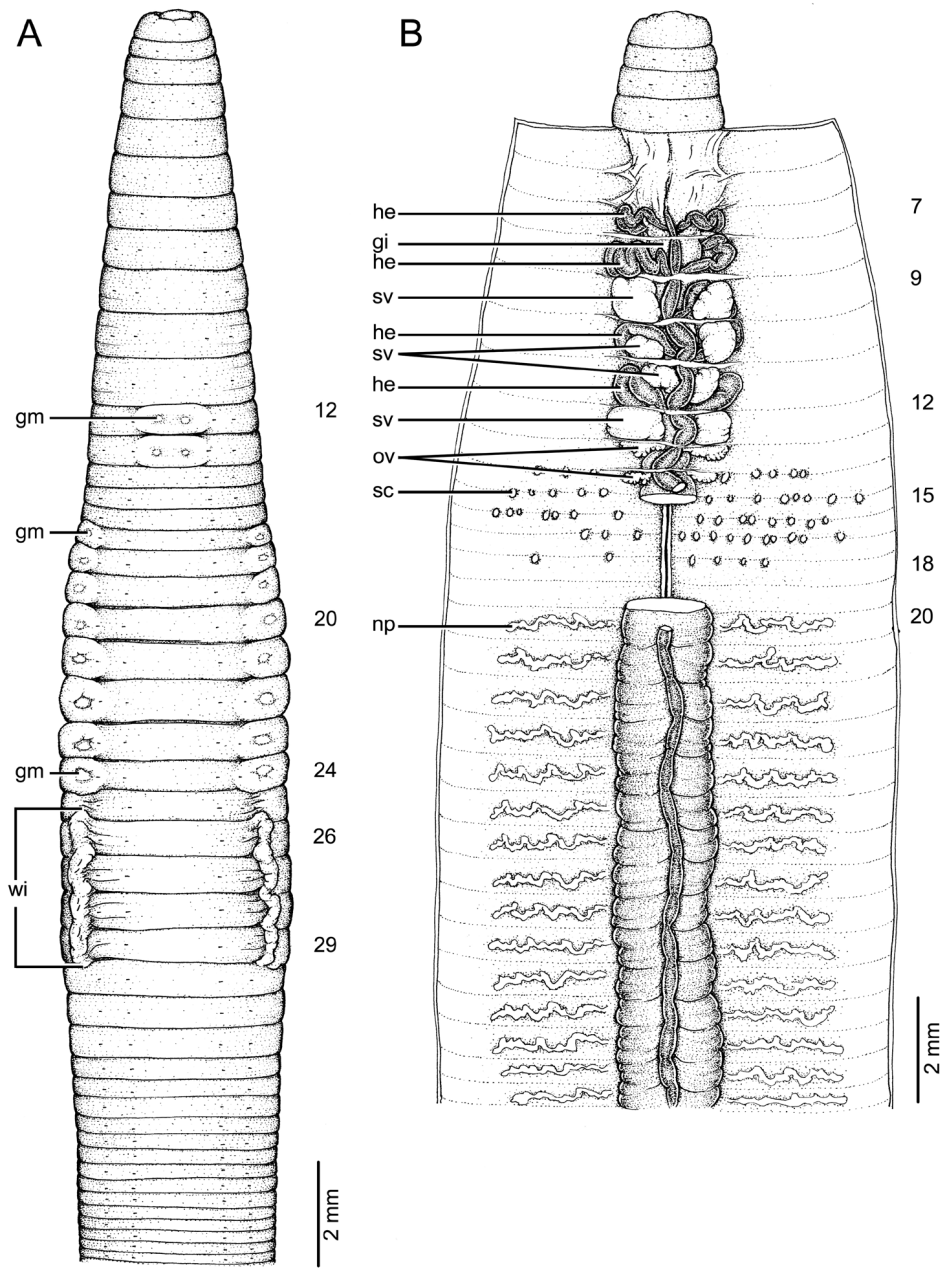
Morphology of holotype (CUMZ 3403) of *Glyphidrilus nanensis* sp. n. **A** external ventral view, annular clitellum in XVII–XXXIII **B** internal dorsal view.

Figs 1 , 3 , 4 , Table 1

##### Type material.

**Holotype**: One adult (CUMZ 3403) in a rice field near Nan River at Saklek, Phichit, north Thailand (16°30'28.4"N, 100°31'15.0"E), 49 meters elevation on 16 June 2012. **Paratypes**: 42 adults and 10 juveniles (CUMZ 3404), 2 adults (ZMH 14579), 2 adults (NHMUK), and 2 adults (ZRC), all same collection data as holotype.

##### Diagnosis.

A small sized earthworm with the clitellar wings on the lateral side of the body in XXIV, XXV, XXVI– XXVII, XXVIII, XXIX. Clitellum in XVII, XVIII–XXXIII, XXXIV. Female pores, male pores, and spermathecal pores not visible. Genital markings: paired or asymmetrical on aa in X, XI, XII, XIII, XIV and XXVIII, XXIX, XXX; paired or asymmetrical on bc in XV, XVI, XVII–XXIII, XXIV, XXV. Four pairs of seminal vesicles in IX–XII. Intestinal origin in XV. Ovaries in XIII–XIV. Spermathecae between 13/14–17/18.

##### Description of holotype.

Dimensions: body length 78 mm, diameter 2.6 mm in segment VIII, 4.7 mm before the clitellar wing in segment XXIII, 4.2 mm after wing in segment XXX in clitellar region; body cylindrical in anterior part, quadrangular in transverse section behind clitellum. 241 segments. Body color pale brown with variations from red to pink at adjacent tissues of wing portion in different individuals of newly collected specimens. At posterior end dorsal surface considerably broader than the ventral. Clitellar wing on ventro-lateral part of clitellum in XXVI–XXIX, 3.6 mm in height, and 0.3 mm in width on both sides. Prostomium zygolobous. Dorsal pores absent. Clitellum annular in XVII–XXXIII. Four pairs of setae per segment from II, setal formula aa:ab:bc:cd:dd = 1.0:0.6:1.3:0.6:1.4 in segment VIII and 1.0:0.6:1.5:0.6:1.5 in postclitellar segments. Female pores, male pores, and spermathecal pores not visible. Genital markings: paired on aa in XII–XIII, laterally paired or asymmetrical on bc in XVII–XXIV.

Septa 5/6–8/9 thicker than between other segments, 9/10–14/15 thick and 15/16 to the last segment thin. Gizzard small, globular in 7/8. Intestine enlarged from XV. Hearts, five pairs in VII–XI, first in VII and last in XI. A pair of holonephridia in each segment from segment XIII onwards. Seminal vesicles, four pairs in IX, X, XI, XII. Ovaries, two pairs in XIII–XIV. Testes free in X and XI. Prostate and accessory glands absent. Spermathecae sessile and diverticula absent, elongated oval or globular between 13/14–17/18, about 0.2–0.3 mm in diameter, two to ten on each side per segment.

##### Variations.

Body lengths of adult (n = 49) and juvenile paratypes (n = 10). Adults ranged from 72–99 mm (85.2±19.0), with 186–257 segments. Juveniles ranged from 73–93 mm (82.3±7.5), with 186–251 segments. Wings in XXIV, XXV, XXVI–XXVII, XXVIII, XXIX, clitellum in XVII, XVIII –XXXIII, XXXIV. Genital markings: paired or asymmetrical on aa in X, XI, XII, XIII, XIV and XXVIII, XXIX, XXX; paired or asymmetrical on bc in XV, XVI, XVII–XXIII, XXIV, XXV.

##### Distribution.

The new species was found in the river banks of Nan River at Saklek, Phichit, north Thailand, which was covered with worm casts.

##### Etymology.

The species was named after the locality on the banks of the Nan River. This was the first time that the worm genus was ever recorded from near this river.

##### Remarks.

For a summary of the following comparisons please see Table 1. The new species differs from *G. yunnanensis* Chen & Xu, 1977 reported from China by the latter having longer wings in XXII–XXXII, the clitellum in XVIII–XXXVIII, paired genital markings on bc in XVII–XXI and XXXII–XXXIV, lacking a spermathecae. It differs from *G. vangviengensis* Chanabun & Panha, 2011 from Laos by *G. vangviengensis* having longer wings in XXIV, XXV–XXXI, XXXII, the clitellum in XIX, XX–XXXV, XXXVI, XXXVII, and lacking spermathecae. It differs from the species recorded as *G. mekongensis* Panha & Chanabun, 2012 from the Mekong River, Ubon Ratchathani, northeast Thailand by the latter species having longer wings in XXIV–½XXXIII, XXXIII, XXXIV, ½XXXV, the clitellum in XIX–XXXVII, XXXVIII, and lacking spermathecae. It differs from *G. borealis* Chanabun & Panha, 2013 from Chiangmai, north Thailand by the latter having longer wings in XXI, XXII–XXVII, XXVIII, XXIX, a longer clitellum in XIV, XVI, XVII–XXXI, XXXII, XXXIII, XXXIV, XXXV, XXXVI, and the intestine enlarged from XIII. It differs from *G. vangthongensis* Chanabun & Panha, 2013 reported from Phitsanulok, north Thailand by *G. vangthongensis* having longer wings in XXIV, XXV, XXVI–XXXI, XXXII, a longer clitellum in XII, XIII, XIV, XV, XVI–XL, XLI, XLII, an intestine enlarged from XIV, and spermathecae between 12/13–18/19. It differs from *G. chaophraya* Chanabun & Panha, 2013 from Chaophraya River, Nakhonsawan, central Thailand by the latter having longer wings in XXIV, XXV–XXXII, XXXIII, a longer clitellum in XX–XLIII, XLIV, XLV, and spermathecae between 16/17–22/23. It differs from *G. chiensis* Chanabun & Panha, 2013 from Mahasarakham, northeast Thailand by *G. chiensis* having wings in XXIII, XXIV, XXV, XXVI–XXIX, XXX, XXXI, XXXII, the clitellum in XVII, XVIII–XXXIII, XXXIV, XXXV, XXXVI, XXXVII, XXXVIII, and spermathecae between 12/13–18/19. It differs from *G. quadratus* Chanabun & Panha, 2013 reported from the Mun River drainage area, northeast Thailand by *G. quadratus* having wings in XXIII, XXIV–XXVIII, XXIX, XXX, XXXI, the clitellum in XV, XVI, XVII, XVIII–XXXI, XXXII, XXXIII, XXXIV, XXXV, XXXVI, and spermathecae between 12/13–17/18. It differs from *G. huailuangensis* Chanabun & Panha, 2013 recorded from Najahlauy National Park, Ubon Ratchathani, northeast Thailand which has wings in XXV, XXVI–XXX, XXXI, a clitellum in XII, XIII, XVI–XXXII, XXXIII, and lacking spermathecae. It differs from *G. sekongensis* sp. n. by *G. sekongensis* sp. n. having longer wings in XXV–XXXI, a longer clitellum in XVI, XVII–XXXVI, XXXVII, and spermathecae between 12/13–15/16 (see Table 1).

**Table 1.** The comparison of characters among *Glyphidrilus* species from Thailand and Laos. The morphological characters of the species described are from type specimens and original description.

Species	Length (mm)	Segments	Cli.	Wi.	Gm.			He.	In.	Gi.	Sc.	Type locality
					Paired on bc	Paired on aa	Un paired on aa					
*G. mekongensis*	125–224	223–382	XIX–XXXVII, XXXVIII	XXIV–½XXXIII, XXXIII, XXXIV, ½XXXV	XXIII	absent	absent	VII–XI	XV	VIII	absent	Khong Chiam, Ubon Ratchathani, Thailand
*G. vangviengensis*	104–160	145–229	XIX, XX–XXXV, XXXVI, XXXVII	XXIV, XXV–XXXI, XXXII	XVIII, XIX, XX, XXI–XXIV, XXXIII, XXXIV	XII–XIV, XV	absent	VII–XI	XVI	VIII	absent	Song River Veintiane, Laos
*G. yunnanensis*	123	139	XVIII–XXXVIII	XXII–XXXII	XVII–XXI, XXXII–XXXIV	absent	absent	VII–XI	XVI	VIII	absent	Yunnan, China
*G. borealis*	66–90	180–284	XIV, XVI, XVII–XXXI, XXXII, XXXIII, XXXIV, XXXV, XXXVI	XXI, XXII–XXVII, XXVIII, XXIX	XIII, XIV, XVI, XVII, XVIII–XXII, XXIII, XXVII, XXVIII, XXIX, XXX	absent	absent	VII–XI	XIII	7/8	14/15–18/19	Maeklang waterfall, Doi Inthanon National Park, Chiangmai, Thailand
*G. vangthongensis*	62–195	150–358	XII, XIII, XIV, XV, XVI– XL, XLI, XLII	XXIV, XXV, XXVI–XXXI, XXXII	XIII, XIV–XXIV, XXV, XXVI, XXXI, XXXII, XXXIII	XII, XIII, XIV, XXX, XXXII, XXXIII, XXXIV, XXXV, XXXVI	absent	VII–XI	XIV	7/8	12/13–18/19	Sakulnothayan waterfall, Vangthong, Phitsanu lok, Thailand
*G. chaophraya*	113–138	325–414	XX–XLIII, XLIV, XLV	XXIV, XXV–XXXII, XXXIII	XVI, XIX, XX–XXIII, XXXII, XXXIII	XII, XIII, XIV, XXXIV, XXXV, XXXVII, XXXVIII	absent	VII–XI	XV	VIII	16/17–22/23	Chao phraya River, Payuha kiri, Nakhonsawan, Thailand
*G. chiensis*	61–193	122–386	XVII, XVIII–XXXIII, XXXIV, XXXV, XXXVI, XXXVII, XXXVIII	XXIII, XXIV, XXV, XXVI–XXIX, XXX, XXXI, XXXII	XV, XVI, XVII, XVIII, XIX–XX, XXI, XXII, XXIII, XXIV, XXX, XXXI, XXXIII	XI, XII, XIII, XIV, XXX, XXXII, XXXIII, XXXIV, XXXV, XXXVI	absent	VII–XI	XV	VIII	12/13–18/19	Rice filed at Thatoom, Mueang, Mahasa rakham, Thailand
*G. quadratus*	54–156	186–378	XV, XVI, XVII, XVIII–XXXI, XXXII, XXXIII, XXXIV, XXXV, XXXVI	XXIII, XXIV–XXVIII, XXIX, XXX, XXXI	XIII, XV, XVI, XVII, XVIII, XIX–XXI, XXII, XXIII, XXX, XXXI	XI, XII, XIII, XIV, XXXI, XXXII, XXXIII, XXXIV	absent	VII–XI	XV	VIII	12/13–17/18	Kang Sapue, Phibonmang sahan, Ubon Ratchathani, Thailand
*G. huailuangensis*	50–91	131–228	XII, XIII, XVI–XXXII, XXXIII	XXV, XXVI–XXX, XXXI	XVI–XXIV	XXXI	absent	VIII–XI	XIII	7/8	absent	Huailung waterfall, Najahlauy, Ubon Ratchathani, Thailand
*G. trangensis*	11^+^–63^+^	41^+^–153^+^	XVII, XVIII–XXX	XXII, XXIII–XXVII, XXVIII	absent	absent	XVIII–XXI	VIII–XI	XVI	8/9	XVIII–XXI	Trang River, Nayong, Trang
*G. wararamensis*	18^+^–120	46^+^–279	XI, XII, XIII–XXXIII, XXXIV, XXXV	XX, XXI–XXVI, XXVII	XIV, XV, XVII–XIX, XX, XXVII	absent	XI–XIII, XIV, XV, XVII, XVIII–XIX, XX, XXVIII, XXIX–XXX	VIII–XI	XIV	6/8	13/14–17/18	Stream near Wattham Wararam, Phanom, Suratthani
*G. kratuensis*	48–93	221–282	XVIII–XXX, XXXI, XXXII	XXIII, XXIV–XXVIII, XXIX, XXX	XIV, XV, XVI, XVII, XVIII, XIX, XXII, XXIII, XXIV, XXIX, XXX	absent	XVII, XIX–XX, XXI, XXII, XXIII, XXX–XXXI, XXXII–XXXIV	VIII–XI	XIV	VIII	14/15–17/18	Kratu waterfall, Kratu, Phuket
*G. nanensis* sp. n.	72–99	186–257	XVII, XVIII–XXXIII, XXXIV	XXIV, XXV, XXVI–XXVII, XXVIII, XXIX	XV, XVI, XVII–XXIII, XXIV, XXV	X, XI, XII, XIII, XIV, XXVIII, XXIX, XXX	absent	VII –XI	XV	7/8	13/14–17/18	Rice field near Nan River, Saklek, Phichit, Thailand
*G. satunensis* sp. n.	60^+^–131	156^+^–326	XVII, XVIII–XXXII, XXXIII, XXXIV, XXXV	XXIV, XXV, XXVI–XXIX, XXX, XXXI	XVII, XVIII, XIX	absent	XVI, XVII, XVIII–XXII, XXIII, XXIV	VIII–XI	XVI	VII	13/14–15/16	Nong Prakpraya, Mueang, Satun, Thailand
*G. chiangraiensis* sp. n.	94–340	89–394	XVII, XVIII, XIX, XX, XXI–XXXVI, XXXVII, XXXVIII, XXXIX	XXIII, XXIV–XXVI, ½XXVII, XXVII, ½XXVIII, XXVIII	XXI, XXII, XXIII, XXIV, XXVII, XXVIII, XXIX–XXXV	XII–XIV, XV, XVI	absent	VII–XI	XVI	VIII	15/16–20/21	Mekong River at Wat Hatkai, Chiang khong, Chiangrai, Thailand
*G. namphao* sp. n.	64–122	190–320	XVII–XXVI, XXIX	XVIII–XXIV	XII–XV, XXV, XXVI	XVII	absent	VII–XI	XVI	VIII	13/14–16/17	Phao River Kamkerd, Bolikhamxai, Laos
*G. sekongensis* sp. n.	90–134	237–337	XVI, XVII–XXXVI, XXXVII	XXV–XXXI	XVI, XVII–XXIV, XXXII	absent	absent	VIII–XI	XVI	VIII	12/13–15/16	Stream at Ban Kiang kong, Lamarm, Sekong, Laos
*G. namdonensis* sp. n.	90–139	183–259	XIX, XX–XXXVI, XXXVII, XXXVIII	XXIV, XXV–XXVIII, XXX	XXII, XXIII, XXIV, XXVII, XXVIII–XXXIII	XIII, XIV	absent	VII–XI	XVI	VIII	14/15–18/19	Done River, Thakhek, Kham mouan, Laos
*G. champasakensis* sp. n.	167–301	248–424	XIX, XX– XLIX, L, LI, LII	XXIII, XXIV–½XXXII, XXXII, XXXIII	XVII, XVIII, XIX–XXII, XXIII, XXIV, XXXII, XXXIII, XXXIV, XXXV	XII, XIII, XXXIII, XXXIV	absent	VII–XI	XVI	VIII	14/15–19/20	Mekong River at Ban Khonkhen, Champa sak, Laos

Abbreviations: Cli.: clitellum; Wi.: wings; Gm.: genital markings; He.: hearts; In.: intestinal caeca; Gi.: gizzard; Sc.: spermathecae.

#### Glyphidrilus satunensis

Taxon classificationAnimaliaOpisthoporaAlmidae

Chanabun & Panha sp. n.

http://zoobank.org/16A9B4B7-9D5F-409E-B438-059A0FC21BB0

**Figure 5.**
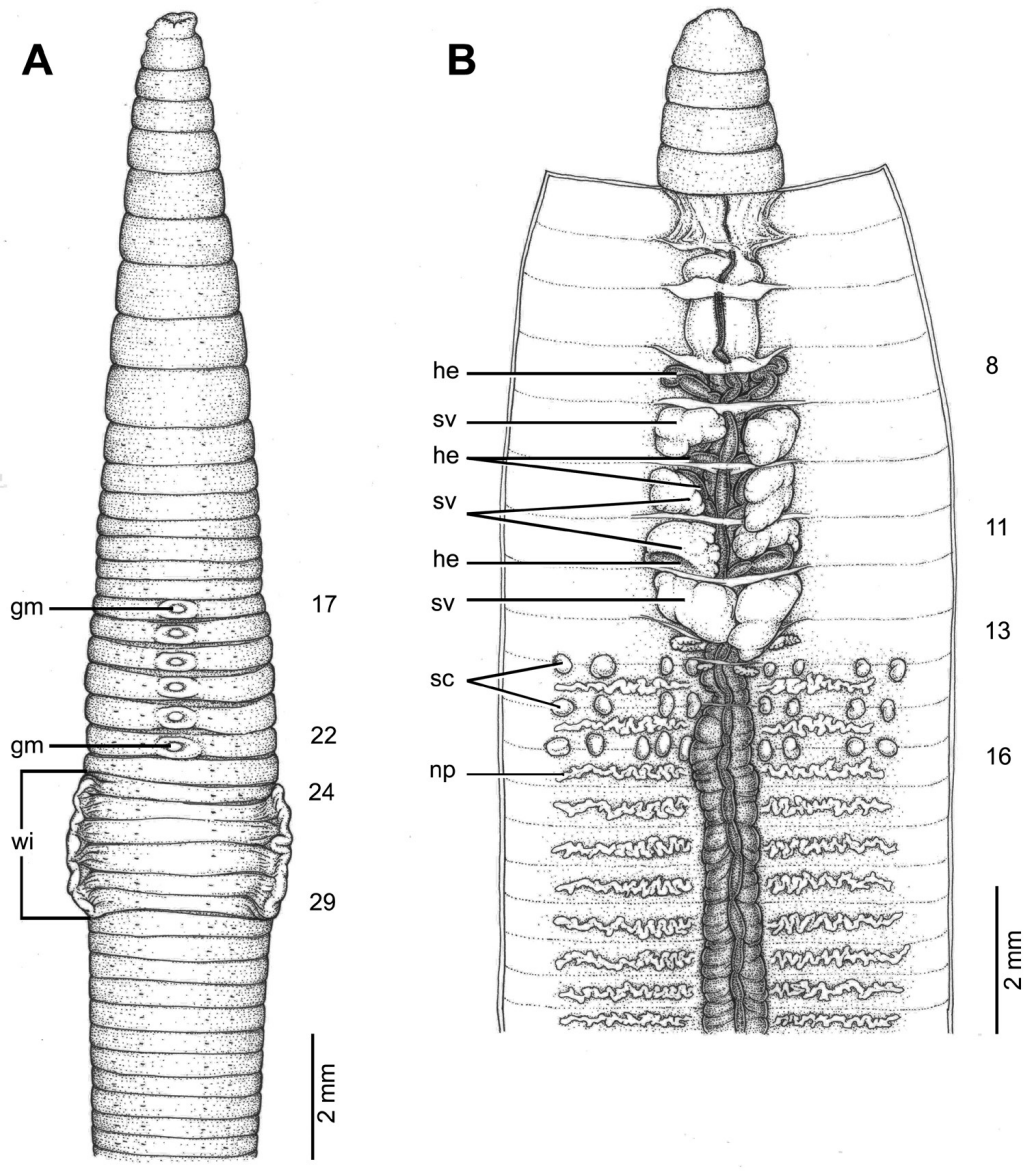
Morphology of holotype (CUMZ 3405) of *Glyphidrilus satunensis* sp. n. **A** external ventral view, annular clitellum in XVII–XXXIII **B** internal dorsal view.

Figs 2 , 5 , Table 1

##### Material examined.

**Holotype**: One adult (CUMZ 3405), in Nong Prakpraya, Mueang, Satun, Thailand (06°44'34.0"N, 100°02'23.0"E), 27 meters elevation on 16 January 2014. **Paratypes**: 15 adults and 21 juveniles (CUMZ 3406), 2 adults (ZMH 14580), 2 adults (NHMUK), and 2 adults (ZRC), all same collection data as holotype.

##### Diagnosis.

*Glyphidrilus satunensis* sp. n. has the clitellar wings on the lateral side of the body in XXIV, XXV, XXVI–XXIX, XXX, XXXI. Clitellum in XVII, XVIII–XXXII, XXXIII, XXXIV, XXXV. Female pores, male pores and spermathecal pores not visible. Genital markings: unpaired on aa in XVI, XVII, XVIII–XXII, XXIII, XXIV; paired or asymmetrical on bc in XVII, XVIII, XIX. Four pairs of seminal vesicles in IX–XII. Intestinal origin in XVI. Ovaries in XIII–XIV. Spermathecae between 13/14–15/16.

##### Description of holotype.

Dimensions: body length 131 mm, diameter 2.2 mm in segment VIII, 2.3 mm before the clitellar wing in segment XXIII, 2.4 mm after wing in segment XXX in clitellar region; body cylindrical in anterior part, quadrangular in transverse section behind clitellum. 326 segments. Body color pale brown with variations from red to pink at adjacent tissues of wing portion in different individuals of newly collected specimens. At posterior end dorsal surface considerably broader than the ventral. Clitellar wing on ventro-lateral part of clitellum in XXIV–XXIX, 2.5 mm in height, and 0.5 mm in width on both sides. Prostomium zygolobous. Dorsal pores absent. Clitellum annular in XVII–XXXIII. Four pairs of setae per segment from II, setal formula aa:ab:bc:cd:dd = 1.0:0.5:1.5:1.0:1.5 in segment VIII and 1.0:0.5:1.5:0.5:2.0 in postclitellar segments. Female pores, male pores, and spermathecal pores not visible. Genital markings: unpaired on aa in XVII–XXII.

Septa 5/6–7/8 thicker than between other segments, 8/9–9/10 thick and 10/11 to the last segment thin. Gizzard small, globular in VII. Intestine enlarged from XVI. Hearts, four pairs in VIII–XI. A pair of holonephridia in each segment from segment XIII onwards. Seminal vesicles, four pairs in IX–XII. Ovaries, two pairs in XIII–XIV. Testes free in X and XI. Prostate and accessory glands absent. Spermathecae sessile and diverticula absent, elongated oval or globular between 13/14–15/16, about 0.2–0.3 mm in diameter, four to five on each side per segment.

##### Variations.

Body lengths of adult (n = 22) and juvenile paratypes (n = 21). Adults ranged from 60^+^–131 mm (95.8 ± 24.5), with 156^+^–326 segments. Juveniles ranged from 62–129 mm (82.5 ± 22.1), with 166–323 segments. Wings in XXIV, XXV, XXVI–XXIX, XXX, XXXI, clitellum in XVII, XVIII–XXXII, XXXIII, XXXIV, XXXV. Genital markings: unpaired on aa in XVI, XVII, XVIII–XXII, XXIII, XXIV; paired or asymmetrical on bc in XVII, XVIII, XIX.

##### Distribution.

The new species was found at a pond in Satun, south Thailand.

##### Etymology.

The name “satunensis” is given in reference to the type locality, Satun.

##### Remarks.

See Table 1 for a summary of these comparisons. *Glyphidrilus satunensis* sp. n. is quite similar to *G. kratuensis* Chanabun & Panha, 2013 from Kratu waterfall, Kratu, Phuket, south Thailand in the location of wings but *G. kratuensis* has a smaller size, a shorter clitellum in XVIII–XXX, XXXI, XXXII, the intestine begins from XIV, and spermathecae are between 14/15–17/18. It differs from *G. trangensis* Chanabun & Panha, 2013 from Trang River, Nayong, Trang, south Thailand by the latter having wings in XXII, XXIII–XXVII, XXVIII, the clitellum in XVII, XVIII–XXX, and spermathecae in XVIII–XXI. It differs from *G. wararamensis* Chanabun & Panha, 2013 from stream near Wattham Wararam, Phanom, Suratthani, south Thailand by *G. wararamensis* having wings in XX, XXI–XXVI, XXVII, a clitellum in XI, XII, XIII–XXXIII, XXXIV, XXXV, and spermathecae between 13/14–17/18. It differs from *G. nanensis* sp. n. by *G. nanensis* having a smaller size, slightly shorter wings in XXIV, XXV, XXVI–XXVII, XXVIII, XXIX, unpaired genital markings absent, five pairs of hearts in VII–XI, and spermathecae between 13/14–17/18 (see Table 1).

#### Glyphidrilus chiangraiensis

Taxon classificationAnimaliaOpisthoporaAlmidae

Chanabun & Panha sp. n.

http://zoobank.org/733E54E7-AE95-451F-998F-BC79E71A4155

**Figure 6.**
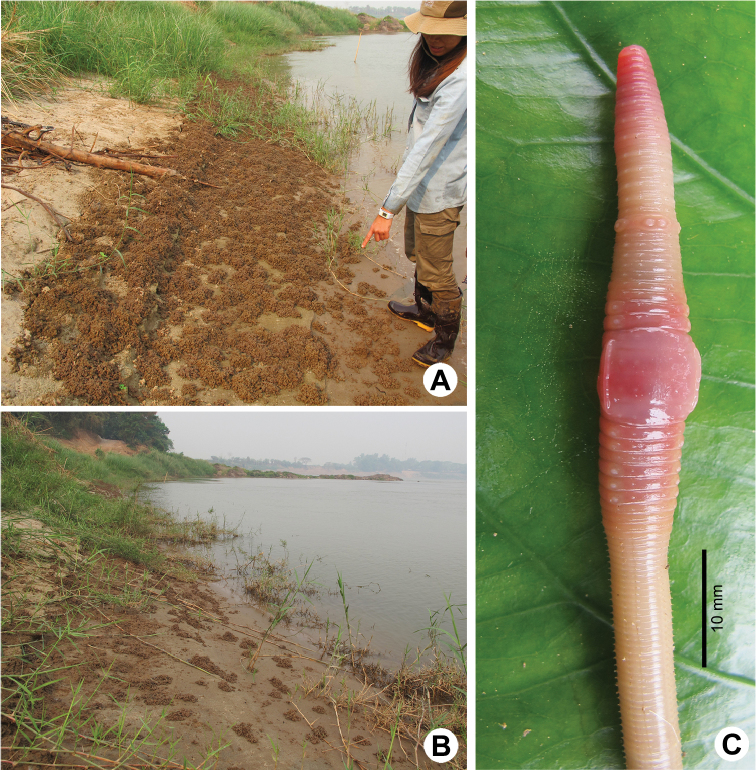
Photographs showing the **A** *Glyphidrilus chiangraiensis* sp. n. and other earthworms casts **B** type locality of *G. chiangraiensis* sp. n. in the river banks of Mekong River at Wat Hatkai, Chiangkhong, Chiangrai, north Thailand, and **C** coloration of newly collected paratype (CUMZ 3408) after the first preservation step in 30% (v/v) ethanol.

**Figure 7.**
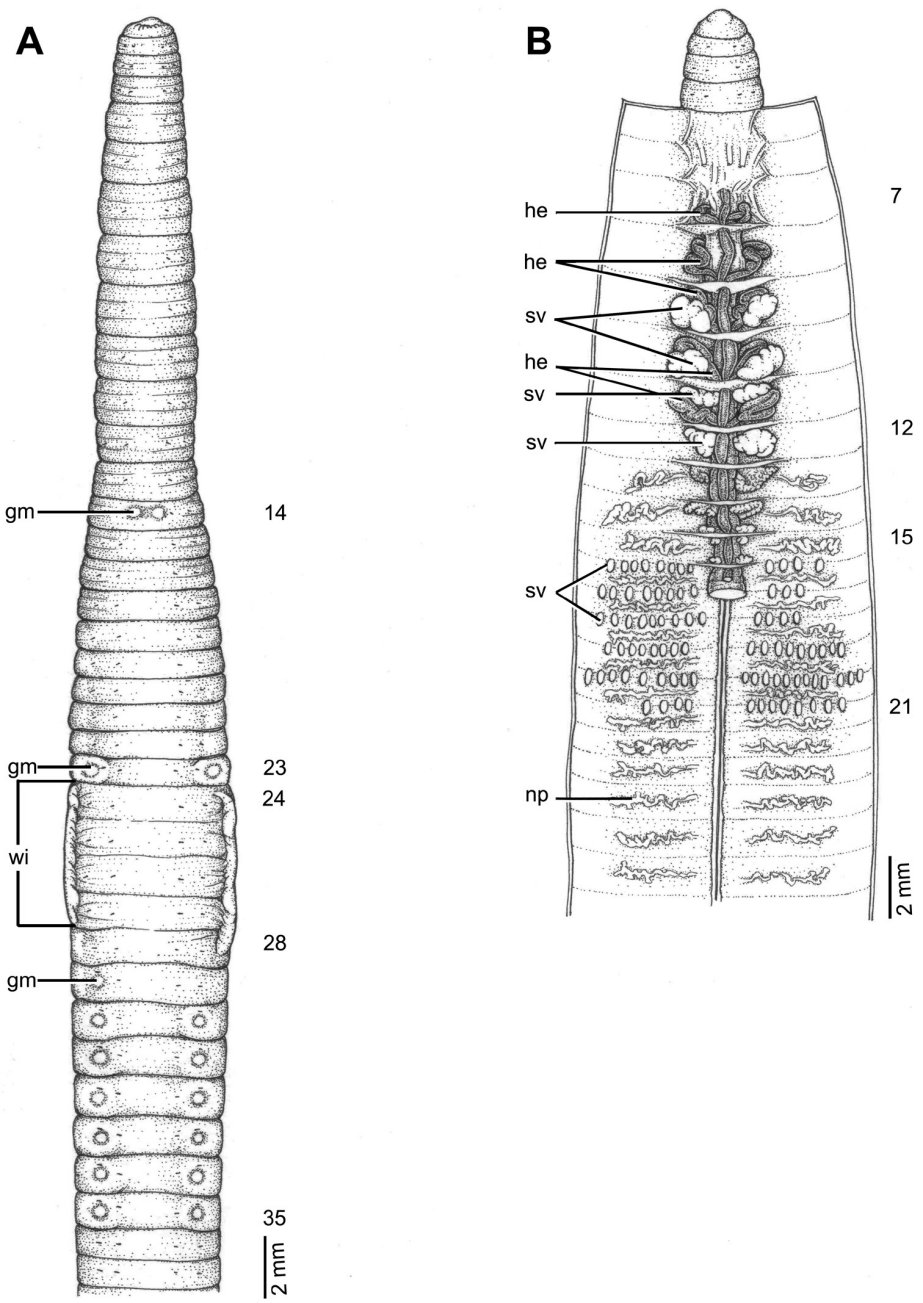
Morphology of holotype (CUMZ 3407) of *G. chiangraiensis* sp. n. **A** external ventral view, annular clitellum in XVII–XXXVIII **B** internal dorsal view.

Figs 1 , 6 , 7 , Table 1

##### Type material.

**Holotype**: One adult (CUMZ 3407) in the river banks of Mekong River at Wat Hatkai, Chiangkhong, Chiangrai, north Thailand (20°15'8.5"N, 100°24'46.8"E), 384 meters elevation on 14 March 2014. **Paratypes**: 9 adults (CUMZ 3408), 2 adults (ZMH 14581), 2 adults (NHMUK), and 2 adults (ZRC), all specimens collected only from the type locality.

##### Other material examined.

43 adults (CUMZ 3409), in the river banks of Mekong River at Mueang, Buengkan, northeast Thailand (18°22'2.4"N, 103°38'58.0"E), 144 meters elevation on 6 December 2013. 33 adults (CUMZ 3410), in the river banks of Mekong River at Kang Kudku, Chiangkhan, Loei, northeast Thailand (17°54'24.5"N, 101°42'7.5"E), 195 meters elevation on 8 December 2013. 25 adults (CUMZ 3411), in the river banks of Mekong River at Wat Srisomsanook, Chiangkhan, Loei, northeast Thailand (17°59'25.4"N, 101°44'51.3"E), 212 meters elevation on 7 December 2013. 11 adults (CUMZ 3412), in the river banks of Mekong River at Wat Hadphatum, Srichiangmai, Nongkhai, northeast Thailand (17°57'32.2"N, 102°35'26.8"E), 174 meters elevation on 7 December 2013. 38 adults (CUMZ 3413), in the river banks of Mekong River at Wat Jomnang, Phonphisai, Nongkhai, northeast Thailand (18°01'53.6"N, 103°4'47.4"E), 165 meters elevation on 6 December 2013. 30 adults (CUMZ 3414), in the river banks of Mekong River at Wat Prayanakmai, Wiangkaen, Chiangrai, north Thailand (20°11'45.2"N, 100°27'32.0"E), 359 meters elevation on 15 March 2014. 19 adults (CUMZ 3415), in the river banks of Mekong River at Wat Bansaw, Chiangsan, Chiangrai, north Thailand (20°15'19.4"N, 100°10'44.9"E), 385 meters elevation on 14 March 2014. 26 adults (CUMZ 3416), in river banks of Mekong River at Ban Rimkhong, Pakchom, Loei, northeast Thailand (18°12'48.9"N, 102°04'52.2"E), 181 meters elevation on 8 December 2013. 3 adults (CUMZ 3417), in river banks of Mekong River, Mueng Paksay, Chaiyaburi, Laos (18°12'40.0"N, 101°24'28.1"E), 214 meters elevation on 15 April 2014. 15 adults (CUMZ 3418) in river banks of Mekong River, Bandon, Luangprabang, Laos (19°55'27.6"N, 102°10'49.7"E), 304 meters elevation on 14 April 2014. 24 adults (CUMZ 3419) in river banks of Mekong River, between Sanakham to Vientiane, Laos (17°57'39.7"N, 101°43'53.8"E), 224 meters elevation on 15 April 2014.

##### Diagnosis.

*Glyphidrilus chiangraiensis* sp. n. has clitellar wings on the lateral side of the body in XXIII, XXIV–XXVI, ½XXVII, XXVII, ½XXVIII, XXVIII. Clitellum in XVII, XVIII, XIX, XX, XXI–XXXVI, XXXVII, XXXVIII, XXXIX. Female pores, male pores and spermathecal pores not visible. Genital markings: paired or asymmetrical on aa in XII–XIV, XV, XVI, paired or asymmetrical on bc in XXI, XXII, XXIII, XXIV and XXVII, XXVIII, XXIX–XXXV. Four pairs of seminal vesicles in IX–XII. Intestinal origin in XVI. Ovaries in XIII–XIV. Spermathecae between 15/16–20/21.

##### Description of holotype.

Dimensions: body length 158 mm, diameter 3.0 mm in segment VIII, 4.0 mm before the clitellar wing in segment XXIII, 4.0 mm after wing in segment XXIX in clitellar region; body cylindrical in anterior part, quadrangular in transverse section behind clitellum. 282 segments. Body color pale brown with variations from red to pink at adjacent tissues of wing portion in different individuals of newly collected specimens. Clitellar wing on ventro-lateral part of clitellum in XXIV–XXVII, ½XXVIII, 3.7 mm and 4.0 mm in height, and 0.5 mm in width on both sides. Prostomium zygolobous. Dorsal pores absent. Clitellum annular in XVII–XXXVIII. Four pairs of setae per segment from II, setal formula aa:ab:bc:cd:dd=2.0:1.0:2.0:1.0:2.0 in segment VIII and 2.0:1.0:2.0:1.0:2.5 in postclitellar segments. Female pores, male pores, and spermathecal pores not visible. Genital markings: paired on aa in XIV, laterally paired or asymmetrical on bc in XXIII and XXIX–XXXV.

Septa 5/6–8/9 thicker than between other segments, 9/10–13/14 thick and 14/15 to the last segment thin. Gizzard small, globular in VIII. Intestine enlarged from XVI. Hearts, five pairs in VII–XI, first in VII and last in XI. A pair of holonephridia in each segment from segment XIII onwards. Seminal vesicles, four pairs in IX, X, XI, XII. Ovaries, two pairs in XIII and XIV. Testis free in X and XI. Prostate and accessory glands absent. Spermathecae sessile and diverticula absent, small elongated oval or globular between 15/16–20/21, about 0.1–0.2 mm in diameter, three to twelve per segment on each side per segment.

##### Variations.

Body lengths of adults (n = 283) ranged from 94–340 mm (155.42 ± 54.93), with 89–394 segments. Wings in XXIII, XXXIV–XXVI, ½XXVII, XXVII, ½XXVIII, XXVIII, clitellum in XVII, XVIII, XIX, XX, XXI–XXXVI, XXXVII, XXXVIII, XXXIX. Genital markings: paired or asymmetrical on aa in XII–XIV, XV, XVI, paired or asymmetrical on bc in XXI, XXII, XXIII, XXIV and XXVII, XXVIII, XXIX–XXXV.

##### Distribution.

The new species is known from the type locality in the river banks of Mekong River at Wat Hatkai, Chiangkhong, Chiangrai, north Thailand, and was found in several locations along the Mekong River and its tributaries in the northeast and north of Thailand at Buengkan, Loei, Nongkhai, and Chiangrai and in Chaiyaburi, Luangprabang, and Sanakham to Vientiane of Laos.

##### Etymology.

The species was named after Chiangrai, the locality name.

##### Remarks.

(see Table 1) *Glyphidrilus chiangraiensis* sp. n. differs from *G. vangviengensis* Chanabun & Panha, 2011 from Song River, Vieintiane, Laos by the latter having longer wings in XXIV, XXV–XXXI, XXXII, the genital markings widely paired in bc XVIII, XIX, XX, XXI–XXIV and XXXIII, XXXIV, paired on aa in XII–XIV, XV and lacking spermathecae. It differs from *G. yunnanensis* Chen & Xu, 1977 reported from China by *G. yunnanensis* having longer wings in XXII–XXXII, clitellum in XVIII–XXXVIII, and lacking spermathecae. It differs from the species recorded as *G. mekongensis* Panha & Chanabun, 2012 from Mekong River, Thailand by *G. mekongensis* having longer wings in XXIV–½XXXIII, XXXIII, XXXIV, ½XXXV, and lacking spermathecae. It differs from *G. chiensis* Chanabun & Panha, 2013 from Chi River, Mahasarakham, northeast Thailand by *G. chiensis* having longer wings in XXIII, XXIV, XXV, XXVI–XXIX, XXX, XXXI, XXXII, and spermathecae between 12/13–18/19. It differs from *G. quadratus* Chanabun & Panha, 2013 reported from the Mun River by *G. quadratus* having longer wings in XXIII, XXIV–XXVIII, XXIX, XXX, XXXI, a bit longer clitellum in XV, XVI, XVII, XVIII–XXXI, XXXII, XXXIII, XXXIV, XXXV, XXXVI, and spermathecae between 12/13–17/18. It differs from *G. huailuangensis* Chanabun & Panha, 2013 recorded from Najahlauy National Park, Ubon Ratchathani, northeast Thailand by the latter having longer wings in XXV, XXVI–XXX, XXXI, clitellum in XII, XIII, XVI–XXXII, XXXIII, and lacks spermathecae. *Glyphidrilus chiangraiensis* sp. n. differs from *G. namphao* sp. n. by the latter having wings in XVIII–XXIV, clitellum in XVII–XXVI, XXIX, genital markings: paired on aa in XVII; paired or asymmetrical on bc in XII–XV, XXV, XXVI, and spermathecae between 13/14–16/17. It differs from *G. sekongensis* sp. n. by the latter having wings in XXV–XXXI, clitellum in XVI, XVII–XXXVI, XXXVII, and spermathecae between 12/13–15/16. It differs from *G. champasakensis* sp. n. from Mekong River at Ban Khonkhen, Champasak, Laos by the latter having longer wings in XXIII, XXIV–½XXXII, XXXII, XXXIII, longer clitellum in XIX, XX–XLIX, L, LI, LII, and spermathecae between 14/15–19/20.

#### Glyphidrilus namphao

Taxon classificationAnimaliaOpisthoporaAlmidae

Chanabun & Panha sp. n.

http://zoobank.org/D559CDC2-5A05-4D38-93C8-681FB80F3415

**Figure 8.**
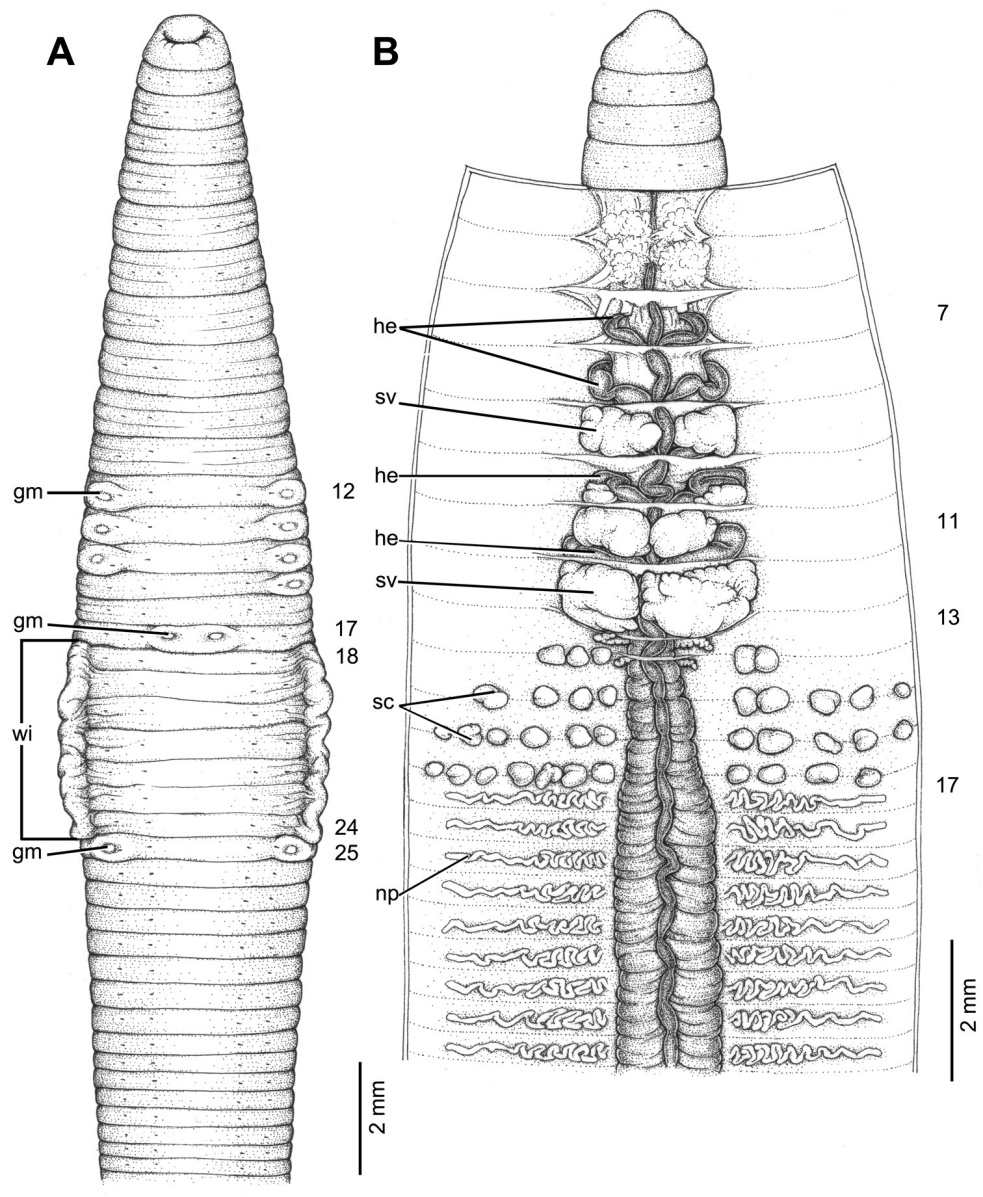
Morphology of holotype (CUMZ 3420) of *Glyphidrilus namphao* sp. n. **A** external ventral view, annular clitellum in XVII–XXIX **B** internal dorsal view.

Figs 1 , 8 , Table 1

##### Type material.

**Holotype**: One adult (CUMZ 3420) in a river banks of Phao River between Ban Lak 7 and Ban Lak 5, Kamkerd, Bolikhamxai, Laos (18°16'27.7"N, 105°2'44.0"E), 525 meters elevation on 7 December 2013. **Paratypes**: 2 adults and 8 juveniles (CUMZ 3421), 1 adult (ZMH 14582), all specimens collected from only the type locality.

##### Diagnosis.

*Glyphidrilus namphao* sp. n. is a small worm with the clitellar wings on the lateral side of the body in XVIII–XXIV. Clitellum in XVII–XXVI, XXIX. Female pores, male pores and spermathecal pores not visible. Genital markings: paired or asymmetrical on aa in XVII; paired or asymmetrical on bc in XII–XV and XXV, XXVI. Four pairs of seminal vesicles in IX–XII. Intestinal origin in XVI. Ovaries in XIII–XIV. Spermathecae between 13/14–16/17.

##### Description of holotype.

Dimensions: body length 92 mm, diameter 3.1 mm in segment VIII, 4.2 mm before the clitellar wing in segment XVII, 4.2 mm after wing in segment XXV in clitellar region; body cylindrical in anterior part, quadrangular in transverse section behind clitellum. 269 segments. Body color pale brown with variations from red to pink at adjacent tissues of wing portion in different individuals of newly collected specimens. At posterior end dorsal surface considerably broader than the ventral. Clitellar wing on ventro-lateral part of clitellum in XVIII–XXIV, 3.5 mm in height, and 0.25 mm in width on both sides. Prostomium zygolobous. Dorsal pores absent. Clitellum annular in XVII–XXIX. Four pairs of setae per segment from II, setal formula aa:ab:bc:cd:dd =1.5:0.5:1.5:0.5:2.0 in segment VIII and 1.5:0.5:1.5:0.5:2.5 in postclitellar segments. Female pores, male pores, and spermathecal pores not visible. Genital markings: paired on aa in XVII, laterally paired or asymmetrical on bc in XII–XV and XXV.

Septa 4/5–6/7 thicker than between other segments, 7/8–11/12 thick and 12/13 to the last segment thin. Gizzard small, globular in VIII. Intestine enlarged from XVI. Hearts, five pairs in VII–XI, first in VII and last in XI. A pair of holonephridia in each segment from segment XIII onwards. Seminal vesicles, four pairs in IX, X, XI, XII. Ovaries, two pairs in XIII and XIV. Testis free in X and XI. Prostate and accessory glands absent. Spermathecae sessile and diverticula absent, elongated oval or globular between 13/14–16/17, about 0.3–0.4 mm in diameter, two to seven on each side per segment.

##### Variations.

Body lengths of adult (n = 4) and juvenile paratypes (n = 8). Adults ranged from 64–122 mm (89.7±20.8), with 190–320 segments. Juveniles ranged from 64–120 mm (98.3±17.6), with 193–311 segments. Wings in XVIII–XXIV, clitellum in XVII–XXVI, XXIX. Genital markings: paired or asymmetrical on aa in XVII; paired or asymmetrical on bc in XII–XV and XXV, XXVI.

##### Distribution.

The new species was found in the river banks of Phao River which was covered with worm casts.

##### Etymology.

The species was named after the Phao River, the type locality.

##### Remarks.

*Glyphidrilus namphao* sp. n. is different from *G. vangviengensis* Chanabun & Panha, 2011 reported from Song River Veintiane, Laos in different locations of wings in XXIV, XXV–XXXI, XXXII, longer clitellum in XIX, XX–XXXV, XXXVI, XXXVII and lacking spermathecae. It differs from *G. nanensis* sp. n. from Nan River at Saklek, Phichit, north Thailand by *G. nanensis* sp. n. having wings in XXIV, XXV, XXVI–XXVII, XXVIII, XXIX, a longer clitellum in XVII, XVIII–XXXIII, XXXIV, and spermathecae between 13/14–17/18. It is different from *G. sekongensis* sp. n. from Ban Kiangkong, Lamarm, Sekong, Laos in the locations of wings in XXV–XXXI, a longer clitellum in XVI, XVII–XXXVI, XXXVII, the genital markings paired or asymmetrical on bc in XVI, XVII–XXIV, XXXII, and spermathecae between 12/13–15/16. *Glyphidrilus namphao* sp. n. differs from *G. chiangraiensis* sp. n. from Mekong River at Wat Hatkai, Chiangkhong, Chiangrai, north Thailand in the locations of wings in XXIII, XXIV–XXVI, ½XXVII, XXVII, ½XXVIII, XXVIII, a longer clitellum in XVII, XVIII, XIX, XX, XXI–XXXVI, XXXVII, XXXVIII, XXXIX, and spermathecae between 15/16–20/21 (see Table 1).

#### Glyphidrilus sekongensis

Taxon classificationAnimaliaOpisthoporaAlmidae

Chanabun & Panha sp. n.

http://zoobank.org/F9F57BA6-C6DC-46EB-8D14-9427E9ADD3E7

**Figure 9.**
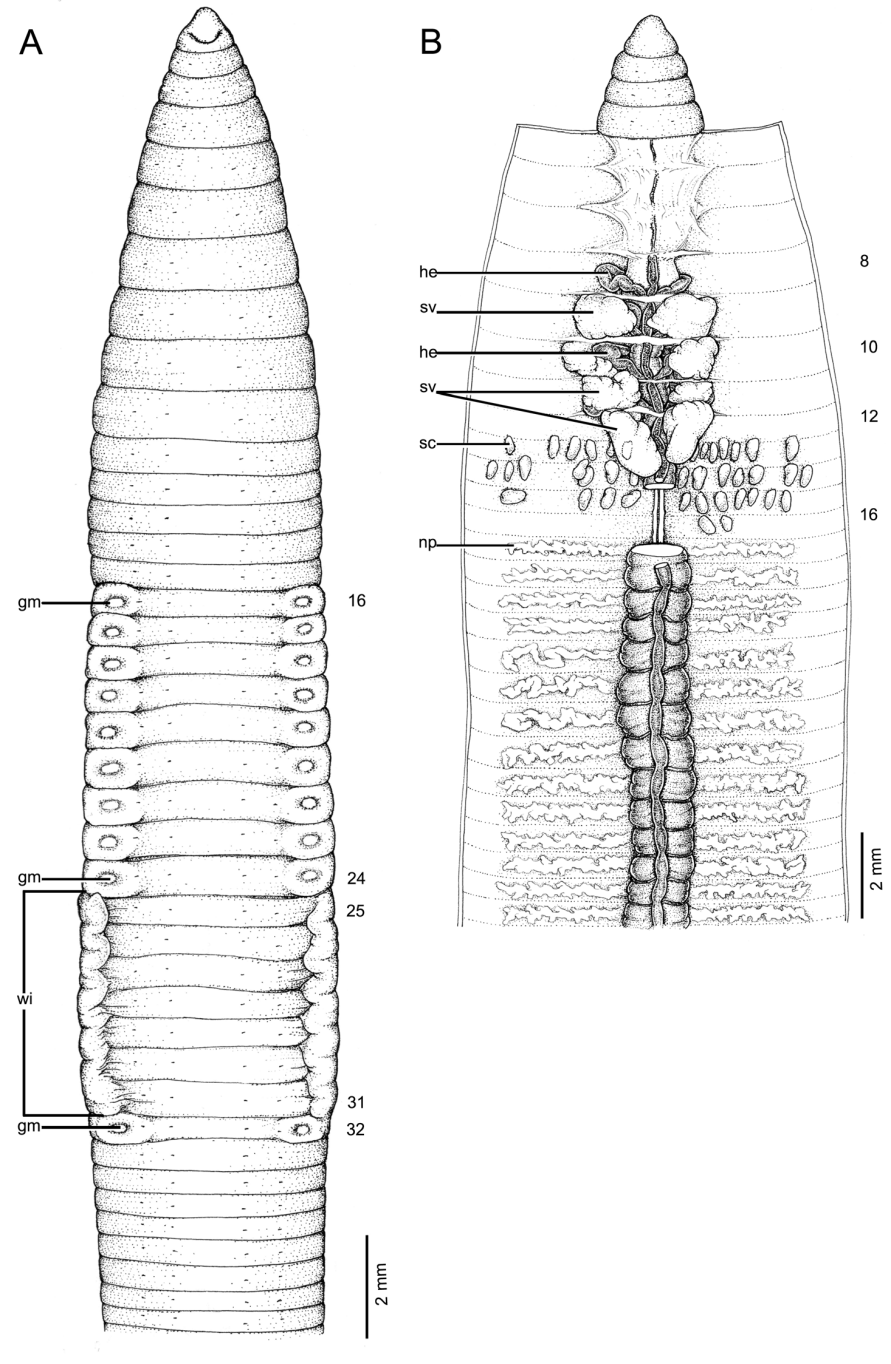
Morphology of holotype (CUMZ 3422) of *Glyphidrilus sekongensis* sp. n. **A** external ventral view, annular clitellum in XVI–XXXVII **B** internal dorsal view.

Figs 1 , 9 , Table 1

##### Type material.

**Holotype**: One adult (CUMZ 3422) in a stream at Ban Kiangkong, Lamarm, Sekong, Laos (15°33'30.6"N, 106°19'19.4"E), 472 meters elevation on 17 October 2013. **Paratypes**: 2 adults and 11 juveniles (CUMZ 3423) all same collection data as holotype.

##### Diagnosis.

*Glyphidrilus sekongensis* sp. n. is a small sized earthworm with the clitellar wings on the lateral side of the body in XXV–XXXI. Clitellum in XVI, XVII–XXXVI, XXXVII. Female pores, male pores and spermathecal pores not visible. Genital markings: paired or asymmetrical on bc in XVI, XVII–XXIV and XXXII. Four pairs of seminal vesicles in IX–XII. Intestinal origin in XVI. Ovaries in XIII–XIV. Spermathecae between 12/13–15/16.

##### Description of holotype.

Dimensions: body length 134 mm, diameter 2.4 mm in segment VIII, 2.3 mm before the clitellar wing in segment XXIV, 2.4 mm after wing in segment XXXII in clitellar region; body cylindrical in anterior part, quadrangular in transverse section behind clitellum. 329 segments. Body color pale brown with variations from red to pink on adjacent tissues of wing portions in different individuals of newly collected specimens. The posterior end on the dorsal surface is considerably broader than the ventral. Clitellar wing on ventro-lateral part of clitellum in XXV–XXXI, 4.0 mm in height, and 0.5 mm in width on both sides. Prostomium zygolobous. Dorsal pores absent. Clitellum annular in XVI–XXXVII. Four pairs of setae per segment from II, setal formula aa:ab:bc:cd:dd = 1.0:0.3:1.0:0.5:1.3 in segment VIII and 1.8:0.8:1.3:0.6:1.5 in postclitellar segments. Female pores, male pores, and spermathecal pores not visible. Genital markings: laterally paired or asymmetrical on bc in XVI–XXIV, XXXII.

Septa 5/6–8/9 thicker than between other segments, 9/10–14/15 thick and 15/16 to the last others segment, 9/10–14/15 thick and 15/16 to the last segment thin. Gizzard small, globular in VIII. Intestine enlarged from XVI. Hearts, four pairs in VIII–XI, first in VIII and last in XI. A pair of holonephridia in each segment from segment XIII onwards. Seminal vesicles, four pairs in IX, X, XI, XII. Ovaries, two pairs in XIII and XIV. Testis free in X and XI. Prostate and accessory glands absent. Spermathecae sessile and diverticula absent, small elongated oval or globular between 12/13–15/16, about 0.1–0.3 mm in diameter, two to seven on each side per segment.

##### Variations.

Body lengths of adult (n = 3) and juvenile paratypes (n = 11). Adults ranged from 90–134 mm (109.6 ± 12.2), with 237–337 segments. Juveniles ranged from 86^+^–130 mm (104.2 ± 14.0), with 209^+^–329 segments. Wings in XXV–XXXI, clitellum in XVI, XVII–XXXVI, XXXVII. Genital markings: laterally paired or asymmetrical on bc in XVI, XVII–XXIV, XXXII.

##### Distribution.

The new species was found in a stream at Ban Kiangkong, Lamarm, Sekong, Laos. Soils are slightly sandy mixed with black organic matter.

##### Etymology.

The species was named after Sekong, the type locality in Laos.

##### Remarks.

(see Table 1). *Glyphidrilus sekongensis* sp. n. differs from other species by the locations of external and internal characteristics such as wings, clitellum, the arrangement of genital markings, and spermathecae. The new species differs from *G. vangviengensis* Chanabun & Panha, 2011 from Song River, Vientiane, Laos by the latter having a shorter clitellum in XIX, XX–XXXV, XXXVI, XXXVII, widely paired genital markings in bc XVIII, XIX, XX, XXI–XXIV, XXXIII, XXXIV, paired on aa in XII, XIII, XIV, XV, and lacks spermathecae. Differences from *G. yunnanensis* Chen & Xu, 1977 reported from China are that *G. yunnanensis* has longer wings in XXII–XXXII, clitellum in XVIII–XXXVIII, and lacks spermathecae. Differences from *G. mekongensis* Panha & Chanabun, 2012 are that *G. mekongensis* has longer wings in XXIV–½XXXIII, XXXIII, XXXIV, ½XXXV, and lacks spermathecae. It differs from *G. huailuangensis* Chanabun & Panha, 2013 recorded from Najahlauy National Park, Ubon Ratchathani, northeast Thailand by the latter having a larger body size, clitellum in XII, XIII, XVI–XXXII, XXXIII, and lacks spermathecae. It differs from *G. champasakensis* sp. n. from Mekong River at Ban Khonkhen, Champasak, Laos by the latter having longer wings in XXIII, XXIV–½XXXII, XXXII, XXXIII, longer clitellum in XIX, XX–XLIX, L, LI, LII, and spermathecae between 14/15–19/20.

#### Glyphidrilus namdonensis

Taxon classificationAnimaliaOpisthoporaAlmidae

Chanabun & Panha sp. n.

http://zoobank.org/64F05048-0871-4BF0-A7F5-B9FB02701348

**Figure 10.**
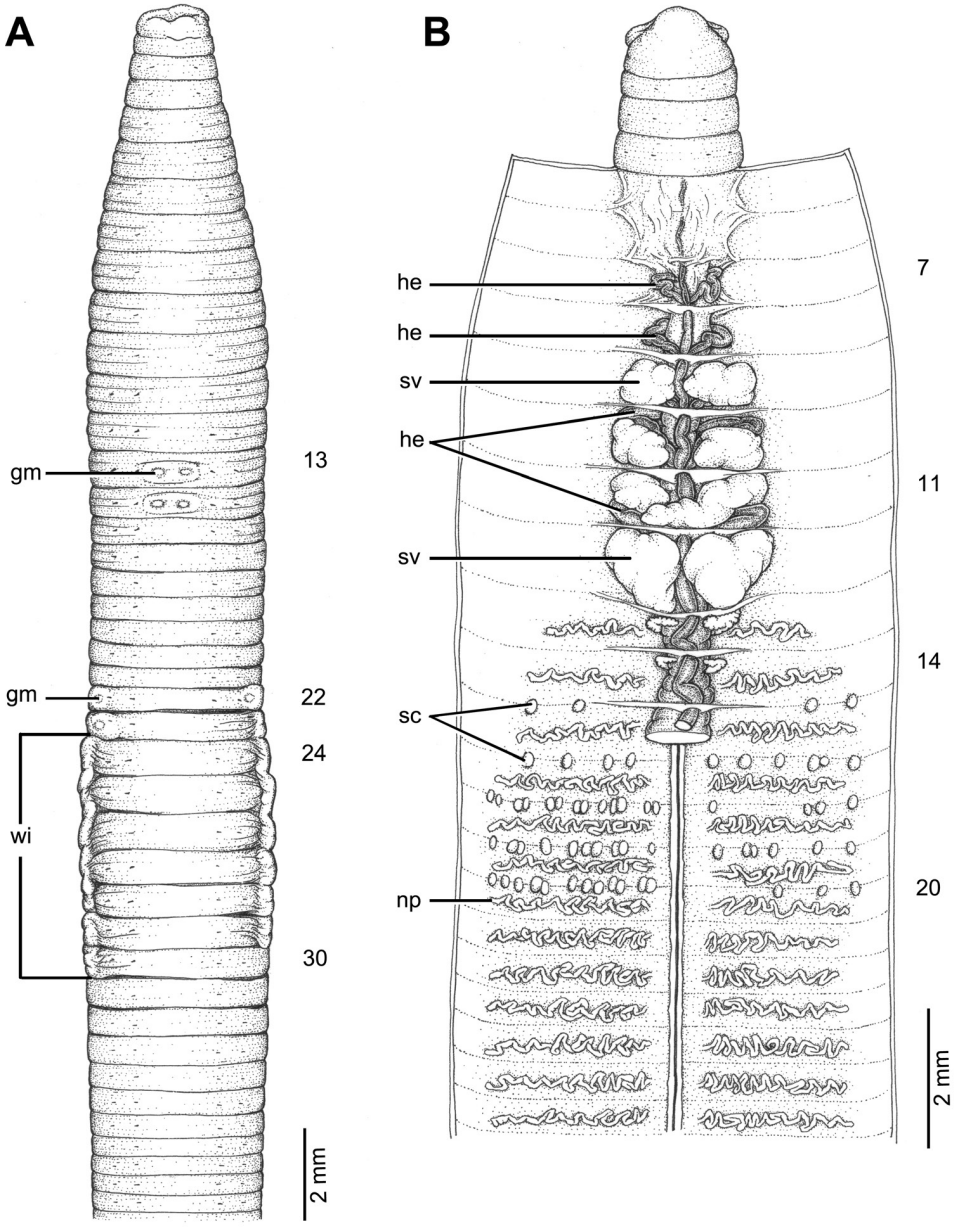
Morphology of holotype (CUMZ 3424) of *Glyphidrilus namdonensis* sp. n. **A** external ventral view, annular clitellum in XX–XXXVII **B** internal dorsal view.

Figs 1 , 10 , Table 1

##### Type material.

**Holotype**: One adult (CUMZ 3424) in the banks of Done River at Ban Namdone, Thakhek, Khammouan, Laos (17°28'39.9"N, 104°45'35.1"E), 161 meters elevation on 6 December 2013. **Paratypes**: 16 adults and 6 juveniles (CUMZ 3425), 2 adults (ZMH 14583), 2 adults (NHMUK), and 2 adults (RMBR), all same collection data as holotype.

##### Diagnosis.

*Glyphidrilus namdonensis* sp. n. is small sized earthworm with distinctly expanded tissues, the clitellar wings on the lateral side of the body in XXIV, XXV–XXVIII, XXX. Clitellum in XIX, XX–XXXVI, XXXVII, XXXVIII. Female pores, male pores and spermathecal pores not visible. Genital markings: medium paired or asymmetrical on aa in XIII, XIV; paired or asymmetrical on bc in XXII, XXIII, XXIV and XXVII, XXVIII–XXXIII. Four pairs of seminal vesicles in IX–XII. Intestinal origin in XVI. Ovaries in XIII–XIV. Spermathecae between 14/15–18/19.

##### Description of holotype.

Dimensions: body length 92 mm, diameter 3.5 mm in segment VIII, 4.0 mm before the clitellar wing in segment XXIII, 4.0 mm after the wing in segment XXXI in the clitellar region; body cylindrical in the anterior part, quadrangular in transverse section behind clitellum. 216 segments. Body color pale brown with variations from red to pink in adjacent tissues of the wing portions in different individuals of newly collected specimens. Clitellar wing on ventro-lateral part of clitellum in XXIV–XXX, 5.0 mm in height, and 0.2 mm in width on both sides. Prostomium zygolobous. Dorsal pores absent. Clitellum annular in XX–XXXVII. Four pairs of setae per segment from II, setal formula aa:ab:bc:cd:dd = 1.3:0.5:1.0:0.5:2.0 in segment VIII and 1.5:0.5:1.0:0.5:2.3 in postclitellar segments. Female pores, male pores, and spermathecal pores not visible. Genital markings: medium paired on aa in XIII, XIV, laterally paired or asymmetrical on bc in XXII, XXIII.

Septa 5/6–8/9 thicker than between other segments, 9/10–14/15 thick, and 15/16 to the last segment thin. Gizzard small, globular in VIII. Intestine enlarged from XVI. Hearts, five pairs in VII–XI, first in VII and last in XI. A pair of holonephridia in each segment from segment XII onwards. Seminal vesicles, four pairs in IX, X, XI, XII. Ovaries two pairs in XIII and XIV. Testis free in X and XI. Prostate and accessory glands absent. Spermathecae sessile and diverticula absent, small elongated oval or globular between 14/15–18/19, about 0.1–0.2 mm in diameter, two to thirteen on each side per segment.

##### Variations.

Body lengths of adult (n = 23) and juvenile paratypes (n = 6). Adults ranged from 90–139 mm (121.9±25.9), with 183–259 segments. Juveniles ranged from 87–136 mm (103.3±17.4), with 185–223 segments. Wings in XXIV, XXV–XXVIII, XXX, clitellum in XIX, XX–XXXVI, XXXVII, XXXVIII. Genital markings: medium paired or asymmetrical on aa in XIII, XIV; paired or asymmetrical on bc in XXII, XXIII, XXIV and XXVII, XXVIII–XXXIII.

##### Distribution.

The new species was found only from the banks of Done River at Ban Namdone, Thakhek, Khammouan, Laos.

##### Etymology.

The species was named after the Done River.

##### Remarks.

*Glyphidrilus namdonensis* sp. n. is compared with other *Glyphidrilus* having spermathecae and a clitellum starting feom segment XIX. *Glyphidrilus namdonensis* differs from *G. champasakensis* sp. n. by the latter having longer wings in XXIII, XXIV–½XXXII, XXXII, XXXIII, longer clitellum in XIX, XX–XLIX, L, LI, LII, and spermathecae between 14/15–19/20 (see Table 1).

#### Glyphidrilus champasakensis

Taxon classificationAnimaliaOpisthoporaAlmidae

Chanabun & Panha sp. n.

http://zoobank.org/32CACB01-68EE-4093-A66E-8EEF939A3B6D

**Figure 11.**
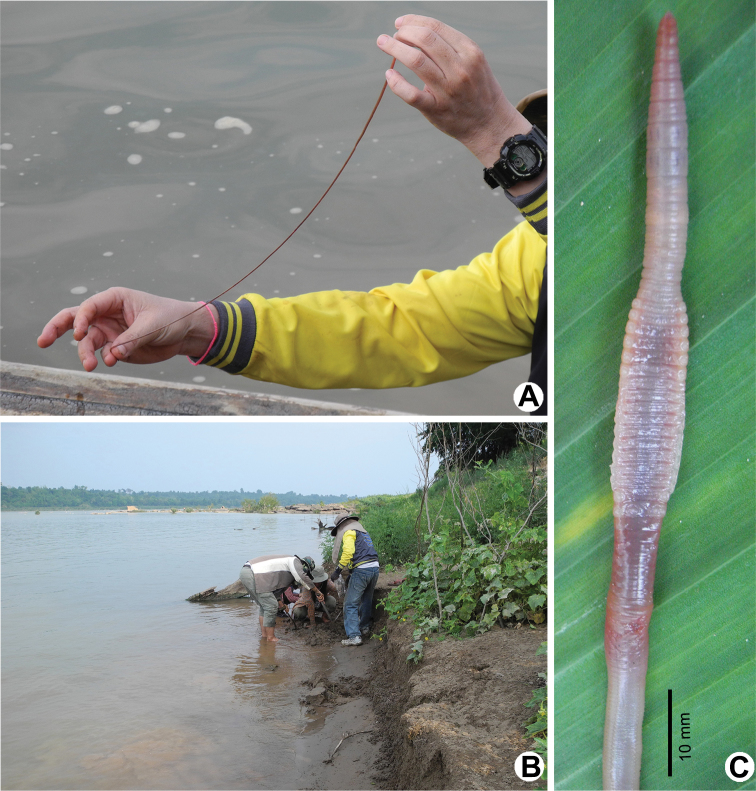
Photographs showing the **A** *Glyphidrilus champasakensis* sp. n. **B** type locality of *G. champasakensis* sp. n. in the banks of Mekong River at Ban Khonkhen, Champasak, Laos and **C** coloration of newly collected paratype (CUMZ 3427) after the first preservation step in 30% (v/v) ethanol.

**Figure 12.**
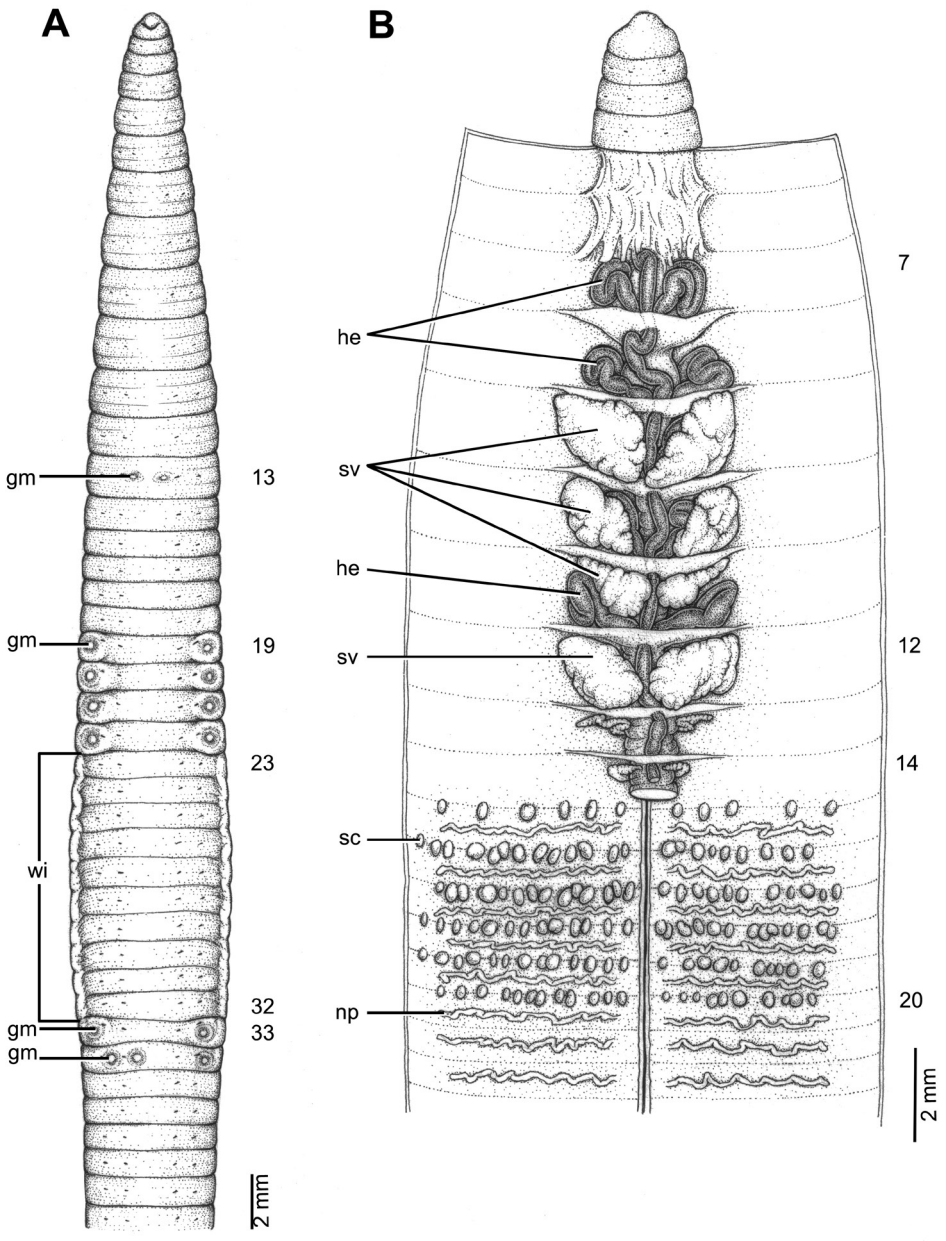
Morphology of holotype (CUMZ 3426) of *Glyphidrilus champasakensis* sp. n. **A** external ventral view, annular clitellum in XIX–XLIX **B** internal dorsal view.

Figs 1 , 11 , 12 , Table 1

##### Type material.

**Holotype**: One adult (CUMZ 3426) in the banks of Mekong River at Ban Khonkhen, Champasak, Laos (15°02'21.2"N, 105°51'20.4"E), 106 meters elevation on 17 April 2014. **Paratypes**: 17 adults (CUMZ 3427), 2 adults (ZMH 14584), 2 adults (NHMUK), and 2 adults (RMBR), all specimens collected from type locality.

##### Diagnosis.

*Glyphidrilus champasakensis* sp. n. is a large sized earthworm with distinct expanded tissues of the clitellar wings on the sides of the body in XXIII, XXIV–½XXXII, XXXII, XXXIII. Clitellum in XIX, XX– XLIX, L, LI, LII. Female pores, male pores and spermathecal pores not visible. Genital markings: medium paired or asymmetrical on aa in XII, XIII, XXXIII, XXXIV; paired or asymmetrical on bc in XVII, XVIII, XIX–XXII, XXIII, XXIV and XXXII, XXXIII, XXXIV, XXXV. Four pairs of seminal vesicles in IX–XII. Intestinal origin in XVI. Large ovaries in XIII–XIV. Spermathecae between 14/15–19/20.

##### Description of holotype.

Dimensions: body length 301 mm, diameter 5.0 mm in segment VIII, 5.0 mm before the clitellar wing in segment XXII, 5.0 mm after wing in segment XXXIII in clitellar region; body cylindrical in anterior part, quadrangular in transverse section behind clitellum. 424 segments. Body color pale brown with variations from red to pink at adjacent tissues of wing portion in different individuals of newly collected specimens. Clitellar wing on ventro-lateral part of clitellum in XXIII–XXXII, 10.0 mm in height, and 0.2 mm in width on both sides. Prostomium zygolobous. Dorsal pores absent. Clitellum annular in XIX– XLIX. Four pairs of setae per segment from II, setal formula aa:ab:bc:cd:dd = 2.0:1.0:2.0:1.0:3.0 in segment VIII and 2.0:1.0:2.0:1.0:2.5 in postclitellar segments. Female pores, male pores, and spermathecal pores not visible. Genital markings: medium paired or asymmetrical on aa in XIII, XXXIV, laterally paired or asymmetrical on bc in XIX–XXII and XXXIII, XXXIV.

Septa 4/5–8/9 thicker than between other segments others segment, 9/10–11/12 thick and 12/13 to the last segment thin. Gizzard small, globular in VIII. Intestine enlarged from XVI. Hearts, five pairs in VII–XI, first in VII and last in XI. A pair of holonephridia in each segment from segment XIV onwards. Seminal vesicles, four pairs in IX, X, XI, XII. Ovaries, two pairs in XIII and XIV. Testis free in X and XI. Prostate and accessory glands absent. Spermathecae sessile and diverticula absent, small elongated oval or globular between 14/15–19/20, about 0.1–0.3 mm in diameter, five to thirteen on each side per segment.

##### Variations.

Body lengths of adult (n = 24) ranged from 167–301 mm (212.75 ± 42.15), with 248–424 segments. Wings in XXIII, XXIV–½XXXII, XXXII, XXXIII, clitellum in XIX, XX– XLIX, L, LI, LII. Genital markings: paired or asymmetrical on aa in XII, XIII, XXXIII, XXXIV; paired or asymmetrical on bc in XVII, XVIII, XIX–XXII, XXIII, XXIV and XXXII, XXXIII, XXXIV, XXXV.

##### Distribution.

The new species was found only on the banks of the Mekong River at Ban Khonkhen, Champasak, Laos.

##### Etymology.

The new species was named after Champasak, Laos, the type locality.

##### Remarks.

Here we compare *Glyphidrilus champasakensis* sp. n. to the species with spermathecae and a clitellum beginning from segment XIX. It differs from *G. namdonensis* sp. n. by *G. namdonensis* sp. n. having shorter wings in XXIV, XXV–XXVIII, XXX, and spermathecae between 14/15–18/19 (see Table 1).

## Discussion

Semi-aquatic earthworms of the genus *Glyphidrilus* are widely recorded on the Asian and African continents. Recently, many species have been described from several ecotone areas in Thailand between freshwater and terrestrial habitats of many river basins, where the soil pH varies from neutral to basic at 7–7.5 (Chanabun et al. 2013).

The seven new species from Thailand and Laos presented in this paper range in size, with respect to the other *Glyphidrilus* members, from large to small, with *G. champasakensis* sp. n. being the longest and *G. nanensis* sp. n. the smallest. The other five species are almost of the same size, as shown in Table 1. However, the locations of clitellum, wings, and spermathecae and other characters of the seven species show clear differences from the closely related species.

**Figure 13.**
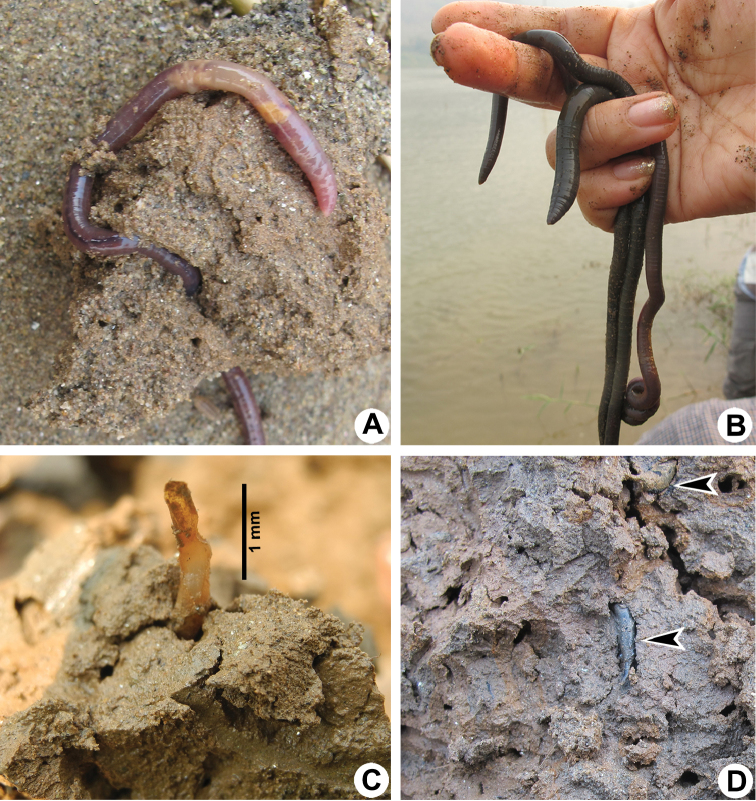
Photographs showing **A** *Metaphire* sp. **B** *Amynthas mekongianus* (Cognetti, 1922) **C** and **D** Cocoons of *Glyphidrilus* in the soil.

*Glyphidrilus chiangraiensis* sp. n. from Mekong River at Wat Hatkai, Chiangkhong, Chiangrai, north Thailand, was found in many areas of the Mekong River and its tributaries from Thailand and Laos, at elevations from 101–385 meters, and co-existing with *G. mekongensis* Panha & Chanabun, 2012, *Amynthas mekongianus* (Cognetti, 1922), *Amynthas* sp., and *Metaphire* sp. The earthworms and cocoons were found in the soil when digging (Fig. 13) and this is probably the cause of the wide distribution, since cocoons travelled from the high to low elevations during the rainy season. *Glyphidrilus nanensis* sp. n. was found in the rice fields of a tributary of the Nan River, in north Thailand. It was found co-existing with the common terrestrial earthworm *Drawida beddardi* (Rosa, 1890). *Glyphidrilus nanensis* sp. n. is probably an isolated endemic occurring in the Nan River valley. This earthworm was found at soil depths of 5–10 cm in agricultural land. This demonstrates that the species is compatible with at least organic farming and that conservation of the species is not difficult, which is increasingly important in the world. Jouquet et al. (2008b) showed the casts produced by *Glyphidrilus* sp. can be considered as patches of nutrients in paddy ﬁelds. Owa et al. (2003) also observed rice development and greater productivity when earthworm casts were associated to rice plants. Choosai et al. (2010) observed rice development in Northeast Thailand and conﬁrmed the positive effect of earthworms on soil properties, rice yield was higher when presence of casts. This strongly suggests that any agricultural system promoting earthworm development, thereby increasing the number of casts per rice ﬁeld, could be considered as an useful approach for the sustainable management of paddy ﬁelds.

*Glyphidrilus satunensis* sp. n. was found at a pond, part of a wildlife sanctuary at Satun, south Thailand. It has a similar body size with another species described from nearby in the Malay Peninsula (Chanabun et al. 2013); however, the locations of clitellum, wings, and spermathecae show clear differences between the closely related species.

The four new species described from Laos, *G. namphao* sp. n., *G. sekongensis* sp. n., *G. namdonensis* sp. n., and *G. champasakensis* sp. n. live in different habitats. *Glyphidrilus namphao* sp. n. lives in Phao River the river near to the Namphao border between Laos and Vietnam at a higher elevation (525 meters) than the other three new species. *Glyphidrilus sekongensis* sp. n. lives in a stream located within an evergreen and mossy forest at Ban Kiangkong, Lamarm, Sekong; the forest is covers 70% of the area near the stream. The soils are slightly sandy and black with the accumulation of organic matter. *Glyphidrilus namdonensis* sp. n. lives in the sandy banks of the Don River, which is one of the branches of Mekong River at Thathek, Khammouan, Laos. *Glyphidrilus champasakensis* sp. n. is one of the new species along the Mekong River found at a soil depth of 20–40 cm, and has similar characteristics to two other new species (*G. sekongensis* sp. n. and *G. namdonensis* sp. n.).

The following key includes only the species most closely related to those described here. The basis for including the species covered in the key is the same as the basis for including previously described species for comparison in the Remarks sections. The remaining species of *Glyphidrilus* must be identified with other resources.

### Key to species of the newly described species of *Glyphidrilus*, and those most similar to them

**Table:** 

1	Spermathecae absent	**2**
–	Spermathecae present	**5**
2	Wings in XXII–XXXII, and clitellum in XVIII–XXXVIII	***G. yunnanensis* Chen & Xu, 1977**
–	Wings from XXIV or XXV	**3**
3	Clitellum in XII, XIII, XVI–XXXII, XXXIII, wings in XXV, XXVI–XXX, XXXI	***G. huailuangensis* Chanabun & Panha, 2013**
–	Clitellum from XIX or XX	**4**
4	Wings in XXIV–½XXXIII, XXXIII, XXXIV, ½XXXV, clitellum in XIX–XXXVII, XXXVIII, and intestine beginning from XV ***G. mekongensis* Panha & Chanabun, 2012**
–	Wings in XXIV, XXV–XXXI, XXXII, clitellum in XIX, XX–XXXV, XXXVI, XXXVII and intestine beginning from XVI	***G. vangviengensis* Chanabun & Panha, 2011**
5	Heart in VII–XI	**6**
–	Heart in VIII–XI	**15**
6	Wings in XVIII–XXIV, clitellum in XVII–XXVI, XXIX, and spermathecae between 13/14–16/17	***G. namphao* sp. n.**
–	Wings beginning from XX	**7**
7	Gizzard beginning from 7/8	**8**
–	Gizzard beginning in segment VIII	**10**
8	Wings in XXI, XXII–XXVII, XXVIII, XXIX, clitellum in XIV, XVI, XVII–XXXI, XXXII, XXXIII, XXXIV, XXXV, XXXVI and spermathecae between 14/15–18/19	***G. borealis* Chanabun & Panha, 2013**
–	Wings beginning from XXIV or XXV or XXVI	**9**
9	Wings in XXIV, XXV, XXVI–XXXI, XXXII, clitellum in XII, XIII, XIV, XV, XVI–XL, XLI, XLII and spermathecae between 12/13–18/19	***G. vangthongensis* Chanabun & Panha, 2013**
–	Wings in XXIV, XXV, XXVI–XXVII, XXVIII, XXIX, clitellum in XVII, XVIII–XXXIII, XXXIV and spermathecae between 13/14–17/18	***G. nanensis* sp. n.**
10	Intestine beginning from XV	**11**
–	Intestine beginning from XVI	**13**
11	Spermathecae between 12/13	**12**
–	Spermathecae between 16/17–22/23, wings in XXIV, XXV–XXXII, XXXIII and clitellum in XX–XLIII, XLIV, XLV	***G. chaophraya* Chanabun & Panha, 2013**
12	Wings in XXIII, XXIV, XXV, XXVI–XXIX, XXX, XXXI, XXXII, clitellum in XVII, XVIII–XXXIII, XXXIV, XXXV, XXXVI, XXXVII, XXXVIII and spermathecae between 12/13–18/19	***G. chiensis* Chanabun & Panha, 2013**
–	Wings in XXIII, XXIV–XXVIII, XXIX, XXX, XXXI, clitellum in XV, XVI, XVII, XVIII–XXXI, XXXII, XXXIII, XXXIV, XXXV, XXXVI and spermathecae between 12/13–17/18	***G. quadratus* Chanabun & Panha, 2013**
13	Spermathecae begin from 14/15	**14**
–	Spermathecae between 15/16–20/21, wings in XXIII, XXIV–XXVI, ½XXVII, XXVII, ½XXVIII, XXVIII and clitellum in XVII, XVIII, XIX, XX, XXI–XXXVI, XXXVII, XXXVIII, XXXIX	***G. chiangraiensis* sp. n.**
14	Wings in XXIV, XXV–XXVIII, XXX, clitellum in XIX, XX–XXXVI, XXXVII, XXXVIII and spermathecae between 14/15–18/19	***G. namdonensis* sp. n.**
–	Wings in XXIII, XXIV–½XXXII, XXXII, XXXIII, clitellum in XIX, XX– XLIX, L, LI, LII and spermathecae between 14/15–19/20	***G. champasakensis* sp. n.**
15	Intestine beginning from XIV	**16**
–	Intestine beginning from XVI	**17**
16	Wings in XX, XXI–XXVI, XXVII, clitellum in XI, XII, XIII–XXXIII, XXXIV, XXXV and spermathecae between 13/14–17/18	***G. wararamensis* Chanabun & Panha, 2013**
–	Wings in XXIII, XXIV–XXVIII, XXIX, XXX, clitellum in XVIII–XXX, XXXI, XXXII and spermathecae between 14/15–17/18	***G. kratuensis* Chanabun & Panha, 2013**
17	Spermathecae intrasegmental in XVIII–XXI, wings in XXII, XXIII–XXVII, XXVIII and clitellum in XVII, XVIII–XXX	***G. trangensis* Chanabun & Panha, 2013**
–	Spermathecae intersegmental in 12/13 or 13/14	**18**
18	Wings in XXIV, XXV, XXVI–XXIX, XXX, XXXI, clitellum in XVII, XVIII–XXXII, XXXIII, XXXIV, XXXV and spermathecae between 13/14–15/16	***G. satunensis* sp. n.**
–	Wings in XXV–XXXI, clitellum in XVI, XVII–XXXVI, XXXVII and spermathecae between 12/13–15/16	***G. sekongensis* sp. n.**

## Supplementary Material

XML Treatment for Glyphidrilus

XML Treatment for Glyphidrilus nanensis

XML Treatment for Glyphidrilus satunensis

XML Treatment for Glyphidrilus chiangraiensis

XML Treatment for Glyphidrilus namphao

XML Treatment for Glyphidrilus sekongensis

XML Treatment for Glyphidrilus namdonensis

XML Treatment for Glyphidrilus champasakensis
